# Knockout of family with sequence similarity 170 member A (*Fam170a*) causes male subfertility, while *Fam170b* is dispensable in mice^†^

**DOI:** 10.1093/biolre/ioaa082

**Published:** 2020-05-22

**Authors:** Darius J Devlin, Kaori Nozawa, Masahito Ikawa, Martin M Matzuk

**Affiliations:** 1 Interdepartmental Program in Translational Biology and Molecular Medicine, Baylor College of Medicine, Houston, TX, USA; 2 Department of Pathology & Immunology, Baylor College of Medicine, Houston, TX, USA; 3 Center for Drug Discovery, Baylor College of Medicine, Houston, TX, USA; 4 Research Institute for Microbial Diseases, Osaka University, Suita, Osaka, Japan; 5 The Institute of Medical Science, The University of Tokyo, Minato-ku, Toyko, Japan

**Keywords:** spermatogenesis, spermatids, sperm, sperm motility and transport, acrosome, testis, epididymis

## Abstract

Families with sequence similarity 170 members A and B (FAM170A and FAM170B) are testis-specific, paralogous proteins that share 31% amino acid identity and are conserved throughout mammals. While previous in vitro experiments suggested that FAM170B, an acrosome-localized protein, plays a role in the mouse sperm acrosome reaction and fertilization, the role of FAM170A in the testis has not been explored. In this study, we used CRISPR/Cas9 to generate null alleles for each gene, and homozygous null (−/−) male mice were mated to wild-type females for 6 months to assess fertility. *Fam170b*^−/−^ males were found to produce normal litter sizes and had normal sperm counts, motility, and sperm morphology. In contrast, mating experiments revealed significantly reduced litter sizes and a reduced pregnancy rate from *Fam170a*^−/−^ males compared with controls. *Fam170a^−/−^*;*Fam170b^−/−^* double knockout males also produced markedly reduced litter sizes, although not significantly different from *Fam170a*^−/−^ alone, suggesting that *Fam170b* does not compensate for the absence of *Fam170a*. *Fam170a*^−/−^ males exhibited abnormal spermiation, abnormal head morphology, and reduced progressive sperm motility. Thus, FAM170A has an important role in male fertility, as the loss of the protein leads to subfertility, while FAM170B is expendable. The molecular functions of FAM170A in spermatogenesis are as yet unknown; however, the protein localizes to the nucleus of elongating spermatids and may mediate its effects on spermatid head shaping and spermiation by regulating the expression of other genes. This work provides the first described role of FAM170A in reproduction and has implications for improving human male infertility diagnoses.

## Teaser

The testis-specific protein FAM170A plays a role in sperm head morphology and motility that is critical for fertility, while its paralog, FAM170B, is not essential.

## Introduction

Infertility is a condition that affects an estimated 15% of couples globally, and of which, nearly half of these cases are attributed to a male factor [1, 2]. Although many issues with male infertility can be overcome by in vitro fertilization (IVF), nearly one-third of male infertility cases are idiopathic with potentially unidentified genetic links [2]. Gene knockout (KO) mouse models have been instrumental in discovering many proteins that are essential to fertility in mammals, thus identifying new genes to improve infertility diagnoses and validating potential targets for contraceptive development. As reproduction is such an important process for the survival of species, it is understandable that functional redundancy in many proteins involved in reproduction acts as a natural layer of protection. We have recently reported 84 testis-specific genes [3, 4] and 9 epididymis-specific genes [5] that were found to be expendable for male fertility using KO mouse models. As nearly 4% of the mouse genome is dedicated to male reproductive tract-specific genes [6], an overarching goal of the field is to determine which of these genes are essential to make inferences about the molecular regulation of human male fertility.

Asthenozoospermia, or reduced sperm motility, is a condition of male infertility resulting from the requirement of vigorous progressive motility for sperm to traverse the female reproductive tract and to penetrate the cumulus-oocyte complex for fertilization [7, 8]. Normally, as a sperm travel through the female reproductive tract environment, many molecular changes are made which give the sperm fertilizing capacity (termed capacitation) and allow sperm to achieve the hyperactivated motility needed to penetrate the oocyte’s rigid zona pellucida glycoprotein matrix [9, 10]. We have previously reported on genes whose KO led to asthenozoospermia, such as Tektin 3 (TEKT3) [11], Tektin 4 (TEKT4) [12], T-complex-associated-testis-expressed 1 (TCTE1) [13], and T-complex 11 (TCP11) [14], with each having unique roles in sperm motility. These studies have contributed to our understanding of the molecular mechanisms governing spermatogenesis, sperm motility, and male fertility.

In this report, we describe the role of a new essential testis-specific gene, Family with sequence similarity 170 member A (*Fam170a*, previously Znfd) in spermiogenesis, as well as the expendability of its paralog, *Fam170b*, using KO mouse models. FAM170A was discovered in reports by Lei [15] and Xu [16], where recombinantly expressed human FAM170A localized to the nucleus of HeLa cells and was shown to have transcriptional activator activity in COS-7 cells. FAM170B was discovered in a report by Li [17], where an anti-FAM170B antibody resolved the protein’s subcellular localization to the acrosome of spermatids and mature sperm. The anti-FAM170B antibody was also used to pretreat mouse sperm before IVF to block FAM170B function, resulting in reduced fertilization. Herein, we evaluate the in vivo significance of the FAM170 proteins in the male reproductive tract using global gene KO mouse models generated by CRISPR/Cas9. We report that the absence of *Fam170a* leads to severe male subfertility characterized by abnormal sperm head morphology, defective spermiation, and reduced sperm progressive motility. In contrast, the absence of *Fam170b* (alone or in combination with *Fam170a* KO) does not impact fertility and is unable to compensate for the loss of *Fam170a*. This work provides the first description of a role for FAM170A in reproduction and definitively determines the requirement of FAM170A and not FAM170B in male fertility.

## Materials and methods

### Animals

All experiments involving animals were approved by the Institutional Animal Care and Use Committee of Baylor College of Medicine (Houston, TX, USA) under protocol AN-716 in the laboratory of M.M.M. and the Animal Care and Use Committee of the Research Institute for Microbial Diseases at Osaka University (Osaka, Japan). All experiments were conducted in compliance with the “Ethical Guidelines and Responsibilities” defined in the Biology of Reproduction Author Guidelines. Mice were maintained under a 12 h:12 h light:dark cycle (from 6:00 AM to 6:00 PM). In this study, C57BL/6J × DBA/2J (B6D2F1) hybrid mice were purchased [Japan SLC (Shizuoka, Japan)] for the generation of mutant mice. Subsequent mutant mice (B6D2 background) were either intercrossed to propagate the colonies or crossed to wild-type (WT) C57BL/6J × 129S6/SvEv (B6129S6) hybrid female mice for fertility experiments.

### Gene and protein bioinformatic analysis

Mouse *Fam170a* (ENSMUSG00000035420) and *Fam170b* (ENSMUSG00000078127) genes were searched in the ENSEMBL genome browser (www.ensembl.org) to identify the comparative genomics gene tree [18, 19]. Protein sequences of representative organisms from unique phylogenetic groups were collected and aligned using Clustal Omega multiple sequence alignment (www.ebi.ac.uk/Tools/msa/clustalo) [20]. Alignment summary graphics were generated using the Jalview program (www.jalview.org/getdown/release) [21]. Protein domain architecture analysis was performed using the SMART protein domain annotation resource (http://smart.embl-heidelberg.de) [22]. Pairwise protein sequence analysis was performed using the SIM alignment tool (https://web.expasy.org/sim) [23]. FAM170A protein subcellular localization in human testis was searched using the Human Protein Atlas database (www.proteinatlas.org/ENSG00000164334-FAM170A/tissue) [24]. Nuclear localization signal (NLS) prediction for FAM170A was performed using the SeqNLS prediction platform (http://mleg.cse.sc.edu/seqNLS) [25].

### RT-PCR and qRT-PCR

Mouse cDNA was prepared from adult (≥8 weeks) C57BL/6J × 129S6/SvEv (B6129S6) hybrid mouse tissues for cross-tissue expression analysis. Total RNA was extracted from each tissue using TRIzol reagent (Invitrogen, Waltham, MA, USA); samples were subjected to DNaseI treatment and RNA clean up with an RNeasy Mini Kit (Qiagen, Hilden, Germany). For RT-PCR, cDNA was synthesized for each tissue using Superscript III reverse transcriptase (Invitrogen). For temporal gene expression analysis, testes were collected from postnatal days (PNDs) 3, 6, 10, 14, 21, 28, 35, and 60 aged pups to prepare cDNA. These time points were selected based on the progression of testis development and the onset of the first wave of spermatogenesis [26]. A 469 bp amplicon for *Fam170a* was amplified using the following primers: (F) 5′-CTCATCCATCGTAGTAAGGGAACC and (R) 5′-CGAGCCAGTCAGGTGTCTTT. A 357 bp amplicon for *am170b* was amplified using the following primers: (F) 5′-GTAGTGAATGTACCAGGCTCC and (R) 5′-CCGATCTCTACTTTCCCTGTTG. Hypoxanthine guanine phosphoribosyl transferase (*Hprt*) was used as an expression control, either amplifying a 164 bp amplicon with the (F) 5′-TACAGGCCAGACTTTGTTGGAT and (R) 5′-ATTTGCAGATTCAACTTGCGCT primer set or a 400-bp amplicon with the (F) 5′-TGGACAGGACTGAAAGACTTGCTCG and (R) 5′-GGCCTGTATCCAACACTTCGAGAGG primer set.

For qRT-PCR, cDNA was synthesized using qScript cDNA Supermix (Quanta Biosciences, Gaithersburg, MD, USA). Primers for all genes assayed were designed to amplify an amplicon smaller than 225 bp (Supplemental Table S1). PCR was carried out in the 2× SYBR Green PCR Master Mix (Invitrogen) in a 10-μl volume within a 384-well plate. Thermocycling was carried out with a Lightcycler 480 (Roche, Basel, Switzerland) with 40 cycles consisting of 15-s denaturation at 95 °C and 1-min annealing/elongation at 60 °C.

### Antibodies

Mouse mAb anti-FLAG (1:1000 dilution, product no. F1804, Sigma, St. Louis, MO, USA), rabbit pAb anti-FLAG (1:100 dilution, product no. F7425, Sigma), rabbit pAb anti-GOLGIN-97 (1:180 dilution, product no. ab84340, Abcam, Cambridge, MA, USA), rabbit pAb anti-TNP1 (1:800 dilution, product no. 17178–1-AP, ProteinTech, Chicago, IL, USA), and mouse mAb anti-α-Tubulin-Alexa Fluor 488 (1:200 dilution, product no. 322588, Invitrogen) were purchased and used for immunostaining. Rabbit pAb anti-FAM170B (1:50 dilution, product no. ab128280, Abcam) was purchased and used for immunostaining as reported by Li [17]. Secondary antibodies donkey anti-mouse IgG-Alexa Fluor 488 (product no. A21202, Life Technologies, Rockville, MD, USA) and donkey anti-rabbit IgG-Alexa Fluor 594 (A21207, Life Technologies) were purchased and used at a 1:500 dilution for immunostaining. Peanut agglutinin conjugated to FITC (PNA-FITC) (1:250 dilution, product no. L-7381, Sigma) was used to label intact acrosomes in mature spermatozoa, and Soybean agglutinin conjugated to FITC (SBA-FITC) (1:400 dilution, product no. FL-1011, Vector Labs, Burlingame, CA, USA) was used to label acrosomes in round and elongating spermatids in the testis, as established in previous reports [27, 28]. 4′,6-Diamidino-2-phenylindole (DAPI) (1-μg/ml dilution, product no. D9542, Sigma) was used for immunostaining to label nuclei.

### Cloning and expression of mFAM170A in COS-7 cells

The full-length mouse FAM170A open reading frame (ORF) was cloned from mouse testis cDNA using forward primer 5′-GCAAGCTTCGCCGCCGCCATGAAACGGCGACAAAAGAGGAAACAT, which contains a HindIII restriction site and Kozak sequence [29], and reverse primer 5′-GCGCGGCCGCTTATTACTTGTCGTCGTCGTCCTTGTAGTCGCCGCCGCCGCTGCCGCTGTTGTCGCAAT, which contains a C-terminal FLAG tag (cFLAG) and NotI restriction site. The mouse FAM170A-cFLAG construct was cloned into the pCAG1.1 mammalian expression vector (sequence available at https://benchling.com/s/seq-K5B2uVbXSISydg4XLINQ/edit) using the HindIII and NotI restriction sites. The pCAG1.1 vector is a modification of the pCAGGS vector previously described by Niwa [30] for enhanced recombinant protein expression in mammalian cells. The resulting plasmid was transfected into COS-7 cells (ATCC CRL-1651) using PEI reagent (product no. 23966-2, Polysciences Inc., Warrington, PA, USA) at a 3:1 PEI to DNA ratio, similar to the method described by Yang [31]. Briefly, 22 × 22 mm glass coverslips were placed into each well of a 6-well plate and COS-7 cells were seeded at 0.5 × 10^6^ cells/well in DMEM media (product no. 11965092, Gibco, Waltham, MA, USA) supplemented with 10% fetal bovine serum and 1% penicillin-streptomycin reagent (product no. 15140122, Gibco) 12 h prior to transfection. For transfection, 3 μg of plasmid DNA was mixed with 9 μg of PEI in serum-free OptiMEM medium (product no. 11058021, Gibco) and allowed to form polymer-DNA complexes at room temperature (RT) for 30 min. The polymer-DNA complex was added dropwise to each well of COS-7 cells, and plates were incubated at 37 °C under 5% CO_2_ for 18 h posttransfection. Media was exchanged to fresh DMEM with supplements after 18 h, and the cells were allowed to express mouse FAM170A protein for up to 72 h posttranfection.

### Immunofluorescence for subcellular localization

For COS-7 cells, cells attached to 22 × 22 mm glass coverslips were incubated for 15 min at RT covered with 4% paraformaldehyde (PFA) in PBS (137 mM NaCl, 2.7 mM KCl, 1.8 mM KH_2_PO_4_, 10 mM Na_2_HPO_4_ pH 7.4) and 0.1% Triton X-100 (PBTx) to fix and permeabilize cells. Coverslips were washed in PBS followed by TBS (20 mM Tris-HCl pH 7.6, 150 mM NaCl) for 5 min each and subsequently blocked for 1 h at RT in 1% skim milk + 1% normal donkey serum (NDS) diluted TBS. Coverslips were briefly washed twice in TBS and then incubated with the primary antibodies anti-FLAG (for FAM170A-cFLAG) and anti-GOLGIN97 (a marker for the trans-Golgi network [32]) diluted in blocking solution overnight at 4 °C in a humidified chamber. Coverslips were washed three times with TBS for 5 min each before incubating with fluorophore-conjugated secondary antibodies diluted in blocking buffer for 1 h at RT. Samples were washed in TBS, followed by incubation with DAPI diluted in TBS for 5 min and a final wash with TBS twice for 5 min each. Coverslips were mounted on slides using Immumount (product no. 1900331, Thermo Fisher Scientific, Waltham, MA USA) and allowed to set overnight at RT before being sealed with nail polish. Slides were imaged with a Zeiss LSM 880 confocal microscope at 63× magnification.

For mouse testis cross sections, tissue from adult mice was dissected and fixed with 4% PFA in PBTx overnight at 4 °C. Testes were incubated in increasing sucrose/PBS buffer from 10 to 20%, allowing the testis to sink to the bottom of each tube before changing buffer. A final incubation in a 1:1 mixture of 20% sucrose:OCT for an overnight incubation at 4 °C was done before placing the tissue in a cryomold, covering with OCT, and freezing the block at −80 °C. The blocks were sectioned at 10 μm and mounted on slides. Tissue sections were blocked with PBTx + 3% BSA + 5% NDS for 1 h at RT. The primary antibody anti-FAM170B or acrosome marker PNA-FITC was diluted in blocking buffer (without Triton X-100) and incubated with sections overnight at 4 °C. Sections were washed with 3% BSA/PBS before incubating with secondary antibodies for 1 h at RT. Sections were washed three times for 10 min each, with DAPI added at 1 μg/ml during the second wash before mounting and sealing slides. Slides were imaged with a Zeiss AxioObserver at 40× magnification.

For mouse spermatozoa, samples were collected from the cauda epididymis or vas deferens and allowed to disperse for 15 min in human tubal fluid (HTF) media (101 mM NaCl, 4.69 mM KCl, 0.2 mM MgSO_4_, 0.37 mM KH_2_PO_4_, 2.04 mM CaCl_2_, 25 mM NaHCO_3_, 2.78 mM Glucose, 0.33 mM sodium pyruvate, 21.4 mM sodium lactate, 75 μg/ml Penicillin G, 50 μg/ml Streptomycin sulfate, and 2 μg/ml phenol red) [33] +9 mg/ml BSA pre-equilibrated in a 37 °C incubator under 5% CO_2_ for 1 h. Sperm from the top portion of the suspension was collected and centrifuged at 200 × *g* for 5 min at 4 °C on a tabletop centrifuge to wash and resuspend the sperm in PBS. Using poly-l-lysine coated slides, 20 μl of sperm suspension was placed on slides and allowed to attach for 1 h at RT. Attached sperm were fixed by incubation with 30 μl of 4% PFA in PBTx for 30 min at RT. Slides were washed in PBS and then blocked with 5% BSA in PBS for 1 h at RT. Primary antibodies were diluted in blocking buffer and incubated with sperm overnight at 4 °C in a humidified chamber. Slides were washed and incubated with fluorescent secondary antibodies diluted in blocking buffer. Slides were washed with PBS, then DAPI diluted in PBS for 10 min, and then a final PBS wash. Slides were mounted with Immumount (Thermo Fisher Scientific), sealed with nail polish, and images with a Zeiss LSM 880 confocal microscope at 40× magnification.

### CRISPR/Cas9-mediated mutant mouse generation

Single guide RNA (sgRNA) target sequences for the *Fam170a* start codon (5′-CCATGAAACGGCGACAAAAG) or the large exon of *Fam170b* (5′-ATGAGTCATCTCCGCGGCCA) were designed using the CRISPRdirect suite (https://crispr.dbcls.jp/), selecting for minimal 12-mer and 8-mer scores to reduce off-target risk [34]. The double-strand break (DSB) efficiency of each sgRNA was tested in vitro using the pX459 and pCAG-EGxxFP validation system, as described previously [35]. Purified crRNA containing the 20-mer target sequences along with the tracrRNA were ordered (Sigma) and assembled into an ribonucleoprotein (RNP) complex with Cas9 protein (Thermo Fisher Scientific) as described previously [5, 36]. Briefly, the RNPs were electroporated into zygotes harvested from superovulated B6D2F1 females using an NEPA21 Super Electroporator (Nepagene, Chiba, Japan). Embryos were cultured overnight to the 2-cell stage before being transferred into the oviducts of pseudopregnant CD-1 (Charles River Labs, Wilmington, MA, USA) mice. Founder mutations in pups born were identified by Sanger sequencing. A genotyping strategy for allele-specific PCR for single nucleotide polymorphisms (SNPs) described by Gaudet [37] was used to genotype the 2-bp deletion mutant allele for *Fam170a* (named *Fam170a^em1Osb^*). Mice were genotyped for *Fam170a^em1Osb^* with the WT forward primer 5′-GACACCATGAAACGGCGACANA or the *Fam170a*^−/−^ forward primer 5′-GACACCATGAAACGGCGACNAG (with a 40-bp tail sequence to differentiate amplicon size) and the common reverse primer 5′-TCTGACAGCATCACATGATAGGCCA. The “N” in the primer sequences signifies nucleotides that were replaced to introduce a mismatch, as suggested by Gaudet [37]. The 35-bp deletion mutant allele for *Fam170b* (named *Fam170b^em2Osb^*) was genotyped with the forward primer 5′-TTTCTCAGGGAAGTTACGCATGG and reverse primer 5′-AGAGTAGGACTGATACTCGGAGG. Genotyping accuracy was confirmed by Sanger sequencing. For both mouse lines, littermates with homozygous WT alleles are referred to as WT, heterozygotes are referred to as +/−, homozygous mutant alleles are referred to as −/−, mice heterozygous at both *Fam170a* and *Fam170b* loci are referred to as double heterozygous (dHET), and mice with homozygous mutant alleles at both loci are referred to as double KO (dKO).

For tissue-relevant subcellular localization of FAM170A, a FLAG epitope tag was introduced to the C-terminus of FAM170A in B6D2F1 zygotes by CRISPR/Cas9 electroporation as described above, using the dual crRNA sequences (5′-AAGGACCATTGCGACAACAG and 5′-TGACAATGCCAGTAGTACAA) targeting the stop codon in exon 4 of the *Fam170a* ORF to maximize efficacy. For electroporation of zygotes, both assembled CRISPR/Cas9 RNP complexes were introduced along with a repair oligo of homology-directed repair (HDR). The single-stranded repair oligo contained a nucleotide sequence encoding a two amino acid spacer followed by the FLAG epitope sequence 5′-GGATCCGATTACAAGGATGACGACGATAAG and flanked by 50-bp homology arms. Founder mutant offspring were verified by PCR and Sanger sequencing. Mice bearing the C-terminal FLAG knock-in (KI) (*Fam170a^cFlag^*) allele were genotyped with the primer set (F) 5′-TCTCAATCTCAGGAAGAAGAAAGGACCA and (R) 5′-ATGTGTCCGACGGAGTGTTGTTC.

### Male fertility assessment

Sexually mature +/− male mice at least 6-weeks old from the *Fam170a* and *Fam170b* mutant lines were continuously caged in trio breeding schemes with two 6-week-old female +/− or −/− litter mates (B6D2 background), while −/− males were mated to WT B6129S6 females for 6 months. During the 6-month mating period, the number of pups born per litter and the number of litters per male each month were recorded. Average litter sizes are presented as the average number of pups per litter from all of the males of each breeding scheme. Average litters/male is presented as the average number of litters sired per male from all males of each genotype.

For timed mating, sexually mature WT or *Fam170a*^−/−^ males were continuously caged with two WT B6129S6 females and each mating was recorded by the presence of a copulation plug. At least 20 plugs in total were counted for both WT and *Fam170a*^−/−^ groups. Pregnancy rates are presented as the average of the number of litters produced divided by the number of plugs counted per male for each genotype. Litter size is presented as the average number of pups produced per plug per male for each genotype.

### Histology and sperm phase contrast

For Periodic Acid-Schiff (PAS)-Hematoxylin staining, whole testis, caput epididymis, and corpus epididymis tissues were collected from ≥8-week-old mice and fixed in Bouin’s fixative (Sigma) for 4 to 6 h while rotating. Tissues were then washed in 70% Ethanol with frequent changes to remove excess fixative. Testes were cut along the transverse plane to allow for cross sectioning. Tissues were submitted in cassettes submerged in 70% ethanol to the Baylor College of Medicine Pathology and Histology Core for processing and paraffin embedding. Embedded tissues were sectioned at 5 μm and mounted on slides. PAS-Hematoxylin staining was conducted according to Ahmed and de Rooij [38], deparaffinized in Histo-Clear (VWR, Radnor, PA, USA), rehydrated from ethanol to water, stained with PAS reagent, counterstained with hematoxylin, dehydrated, cleared with Histoclear, and mounted with permount. Slides were imaged with an Olympus BX41 microscope at 20× magnification for the epididymis and 40× magnification for the testis. For individual spermatogenic stage analysis, slides with whole testis cross sections were scanned at 20× magnification by the Baylor College of Medicine Pathology and Histology Core.

For phase contrast, spermatozoa from the caput or cauda epididymis of adult mice were released by mincing the tissue 30 times with fine scissors in 1.0-ml equilibrated HTF media and incubating for 15 min at 37 °C under 5% CO_2_. The sperm suspension was then diluted 1:50 in 1.0-ml fresh HTF, and a 20-μl sample was placed on a slide with a coverslip. A total of at least 400 individual cauda spermatozoa and at least 150 caput spermatozoa were counted for each genotype, counting at least 100 cauda spermatozoa and at least 40 caput spermatozoa from each mouse sample. The sperm were imaged with an Olympus CKX41 microscope at 20× magnification. Morphological classifications of sperm were made by careful examination of the head, midpiece, and tail with guidance from reports by Oliveira [39], Bruner-Tran [40], and Takeda [41]. For counting testicular sperm, testes from adult mice were removed and decapsulated under a dissection microscope, as described in Kotaja [42], and dark sections of removed seminiferous tubules (in stages VI–VIII) were cut open within a few drops of PBS. The testicular spermatozoa suspension was collected with a wide bore pipette tip, and a 20-μl sample was placed on a microscope slide for observation. At least 80 spermatozoa were observed for each genotype, counting at least 20 spermatozoa from each mouse sample.

### Scanning electron microscopy and scanning transmission electron microscopy

For SEM of spermatozoa, a single cut was made in cauda epididymis tissue and using fine tweezers, fluid released from the epididymis was placed in a 2-ml microcentrifuge tube with 1.0-ml equilibrated TYH media + 4 mg/ml BSA (119.37 mM NaCl, 4.78 mM KCl, 1.19 mM MgSO_4_, 1.19 mM KH_2_PO_4_, 1.71 mM CaCl_2_, 25.07 mM NaHCO_3_, 5.56 mM glucose, 1 mM sodium pyruvate, 75 μg/ml Penicillin G, 50 μg/ml Streptomycin sulfate, and 2 μg/ml phenol red) [43]. Sperm were incubated for 15 min at 37 °C under 5% CO_2_ to allow sperm to disperse and then centrifuged at 300 × *g* for 5 min at RT and resuspended in Dulbecco’s PBS (DPBS) to wash. Sperm were centrifuged again and resuspended in 2.5% glutaraldehyde in DPBS for 30 min at RT to fix. Sperm were washed in increasing concentrations of ethanol from 20 to 100% to dehydrate, incubating for 10 min in each. Sperm were centrifuged at 1500 × *g* for 5 min at RT and resuspended in 50% t-butanol/50% ethanol for 15 min at RT. A 20-μl sample of sperm suspension was dried on a specimen pin mount. The samples were sputter coated with iridium before imaging.

For scanning transmission electron microscopy (STEM) analysis of the testis, adult male mice were anesthetized and perfused with 2.5% glutaraldehyde in PBS by transcardial perfusion, as we recently described [44]. Testes were embedded, prepared for ultrathin sectioning, and mounted by the Houston Methodist Research Institute’s Electron Microscopy Core. Samples were imaged using a Nova NanoSEM 230 (FEI) equipped with a STEM detector.

### Sperm count and motility assessment

Spermatozoa from the cauda epididymis of adult mice (≥12 weeks) were collected by dissecting the tissue and placing it in a 1.5-ml microcentrifuge tube with 1.0-ml HTF media [33] + 9-mg/ml BSA pre-equilibrated in a 37 °C incubator with 5% CO_2_. The tissue was minced by cutting 30 times with fine scissors and placed in the incubator with the tubes ajar under 5% CO_2_ for 15 min to allow sperm to release. Using a wide-bore micropipette tip, sperm was diluted 1:50 into 1.0 ml of fresh equilibrated media. A 20-μl sample of the dilution was placed on a chamber of 100-μm-depth counting slides (CellVision, Heerhugowaard, Netherlands) for measuring with the CEROS II animal CASA machine (Hamilton Thorne, Beverly, MA, USA), and the 1:50 dilution was returned to the incubator. Sperm hyperactivity was measured from the 1:50 dilution after an additional 105 min (120 min total) of incubation to allow capacitation. Hyperactivated sperm were classified using CASAnova (https://uncnri.org/CASAnova), an online multiclass support vector machine algorithm, for detecting sperm motility patterns, that was trained against 2000 manually classified sperm tracks, as described by Goodson [45]. CASAnova uses five kinematic measurements from CASA output files (average path velocity, VAP; curvilinear velocity, VCL; straight line velocity, VSL; amplitude of lateral head, ALH; and beat cross frequency, BCF) to classify sperm into either nonvigorous (slow or weak) or vigorous (progressive, intermediate, or hyperactive) motility patterns. CASAnova was used and analyzed as described previously [44].

### Statistical analysis

Statistical significance between two groups (control and KO) was determined using a two-tailed Student’s *t* test assuming unequal variances with an α level of 0.05. Significance between more than two groups was determined by one-way ANOVA with post hoc Tukey-Kramer HSD test. *P* values less than 0.05 were considered significantly. All statistical analyses were performed using Microsoft Office Excel with the Analysis ToolPak plug-in and the RealStats Resource Pack release 6.8 (www.real-statistics.com, copyright 2013–2020 Charles Zaiontz). Data are represented as means ± SEM.

## Results

### *Fam170a* and *Fam170b* are conserved mammalian testis-enriched genes

**Figure 1:**
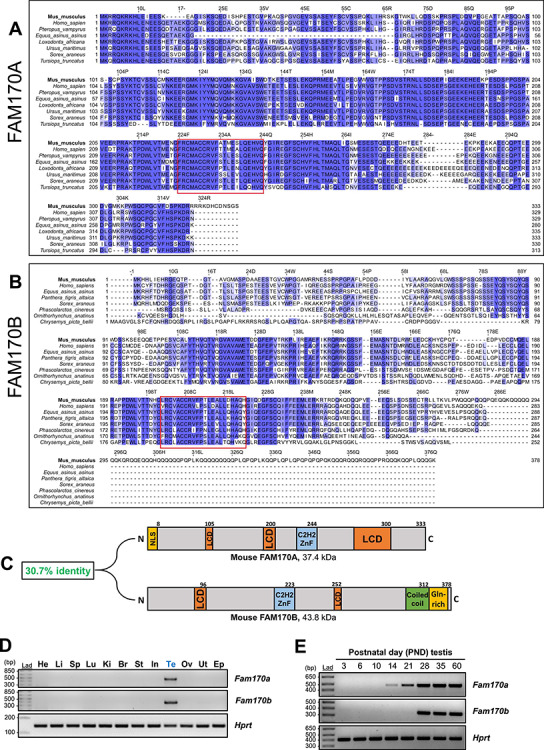
Evolutionary conservation and expression profiles of *Fam170a* and *Fam170b*. Multiple sequence alignment of proteins from representative species for (A) FAM170A and (B) FAM170B. The percentage identity is indicated by the intensity of blue color. The left column contains the taxonomic classification for each organism. The *Mus musculus* sequence has been emboldened as the reference. The red box highlights the C2H2 ZnF domain. (C) Cartoon diagram of FAM170A and FAM170B secondary protein structure showing predicted protein domains and pairwise alignment percent identity score. LCD, low complexity domain; ZnF, zinc finger domain. (D) Cross tissue RT-PCR expression analysis of *Fam170a* and *Fam170b* across 12 mouse tissues. *Hprt* is included as an expression reference control. The 1-kb DNA ladder (Lad) is used as a size marker. (E) RT-PCR temporal expression analysis on testis from WT mice from PNDs 3 to 60.

Multiple sequence alignment for FAM170A and FAM170B proteins across several representative species identified from their respective gene trees revealed that both proteins are well conserved. FAM170A is highly conserved evolutionarily across the eutherian subclass of mammals (Figure 1A), while FAM170B is conserved widely across prototherian, metatherian, and eutherian mammals (Figure 1B). An in silico protein domain analysis detected in both proteins a C2H2 zinc finger domain that is conserved across species (Figure 1A and B, red box), as well as several low complexity domains (Figure 1C). In addition, FAM170B has a predicted coiled-coil domain and a glutamine-rich region at the carboxy-terminus. The FAM170 proteins are paralogs that share 31% identity based on a pairwise sequence analysis (Figure 1C).

In agreement with previous literature [16, 17], the gene expression profiles for *Fam170a* and *Fam170b* were evaluated by multitissue RT-PCR to be testis-specific (Figure 1D) in mice. To determine the onset of expression within the testis, RT-PCR was conducted on testis tissue from WT mice ranging from PNDs 3 to 60 to characterize gene expression from the first wave of spermatogenesis to sexual maturity [46]. Expression of *Fam170a* was detected at PND 14 (Figure 1E), which corresponds to the appearance of pachytene spermatocytes during the meiosis phase of spermatogenesis [26]. In contrast, *Fam170b* shows prominent expression around PND 28 (Figure 1E), well after the first appearance of the round spermatids at PND 18.

### FAM170A and FAM170B exhibit distinct subcellular localization

**Figure 2:**
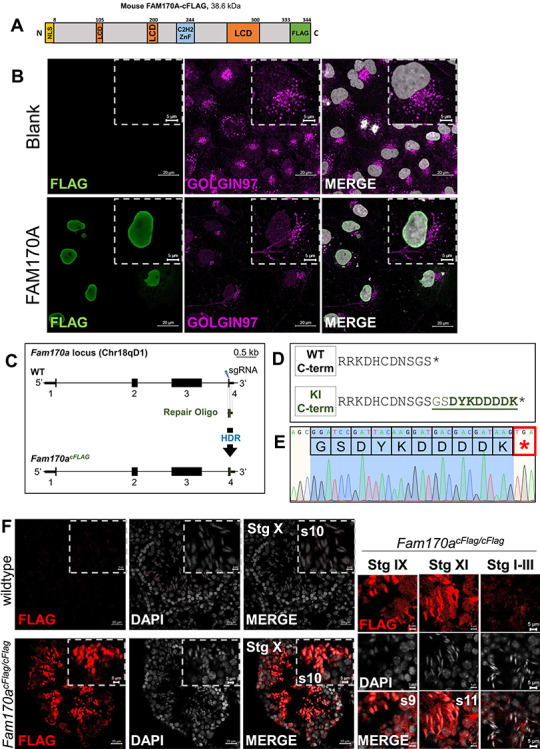
Subcellular localization of C-terminal FLAG tagged FAM170A in COS7 cells and in mouse testis. (A) Cartoon schematic diagram of the recombinant FAM170A construct transfected in COS-7 cells for immunostaining. A C-terminal FLAG epitope tag was added to mouse FAM170A (FAM170A-cFLAG). (B) Immunofluorescent staining of untransfected (Blank) and transfected (FAM170A) COS-7 cells with anti-FLAG antibody to label FAM170A-cFLAG (green), anti-GOLGIN-97 antibody (violet, pseudo colored) to label the trans-Golgi network, and DAPI (gray) to label nuclei. (C) CRISPR/Cas9-mediated targeting of *Fam170a* exon 4 by HDR with a repair oligo containing the nucleotide sequence for the FLAG epitope tag flanked by 50-bp homology arms. The strategy generated the *Fam170a^cFlag^* allele. (D) Comparison of the translated products from exon 4 in WT and *Fam170a^cFlag^* KI mouse alleles. (E) Sanger sequencing confirmation of the *Fam170a^cFlag^* allele from a representative homozygous KI mouse. (F) Immunofluorescent staining of mouse seminiferous tubule cross sections from WT and *Fam170a^cFlag/cFlag^* adult mice with an anti-FLAG antibody (red) and DAPI (gray) counterstaining of nuclei. A strong signal for FAM170A-cFLAG was present in spermatid nuclei in stage IX through stage XI tubules and was absent by stages I–III.

The subcellular localization of FAM170A in the male reproductive tract is unknown, due to the lack of a reliable antibody. Immunohistochemistry data from the Human Protein Atlas, however, show FAM170A localization in the cytoplasm of spermatogonia and within the nucleus of spermatocytes. To confirm this, the full-length mouse FAM170A sequence with a C-terminal FLAG (cFLAG) epitope tag (Figure 2A) was cloned under the CAG promoter in the pCAG1.1 vector and transfected into COS-7 cells. An anti-GOLGIN-97 antibody was used to label the trans-Golgi network in case FAM170A was an acrosome protein, as the acrosome is Golgi-derived organelle [47]. Using an anti-FLAG antibody to label the FAM170A-cFLAG construct and counterstaining with DAPI to label nuclei, FAM170A localized to the nucleus (Figure 2B). These data are in agreement with the previous reports of FAM170A nuclear localization in HeLa cells [15, 16]. Furthermore, FAM170A possesses a strong NLS within the first eight amino acids at the N-terminus, as detected in silico using the SeqNLS database (Supplemental Figure S1). As the endogenous subcellular localization of FAM170A has never been described and in the absence of a reliable commercial antibody for the protein, we used CRISPR/Cas9 to introduce a FLAG tag to the C-terminus of FAM170A in mice by homology-directed repair. A sgRNA targeting the stop codon in exon 4 of *Fam170a* on mouse chromosome 18 was used to introduce a DSB and a repair oligo (with 50-bp homology arms) containing that the nucleotide sequence for a two-amino acid spacer (GS) preceding the FLAG tag sequence was introduced into B6D2F1 zygotes by electroporation (Figure 2C). After culturing the mutant zygotes to the two-cell stage and transferring to the oviducts of pseudopregnant CD-1 females for delivery, mice bearing the intended C-terminal FLAG tag KI allele (Figure 2D) were verified by PCR and Sanger sequencing (Figure 2E). After breeding the FLAG KI to homozygosity (*Fam170a^cFlag/cFlag^*), testes were collected from 12-week-old adult males for immunofluorescent staining with an anti-FLAG antibody. *Fam170a^cFlag/cFlag^* males demonstrated FAM170A localization to the nucleus of elongating spermatids from step 9 (stage IX tubules) to step 11 (stage XI) (Figure 2F). No signal was detected in stages I–III (step 13–step 14 spermatids). Testes from adult WT mice were also stained to confirm the signal specificity. No signal for FAM170A-cFLAG was present in *Fam170a^cFlag/cFlag^* cauda epididymal spermatozoa (data not shown), highlighting that FAM170A is prominent in elongating spermatids in steps 9–11 prior to the condensing phase [48].

In contrast, the subcellular localization for FAM170B was previously reported to be in the acrosome of spermatids and mature spermatozoa [17]. To confirm this, we stained testis cryosections from control adult male mice with an anti-FAM170B antibody and labeled the acrosome with peanut agglutinin (PNA). In agreement with the report by Li [17], FAM170B localized to the acrosome in spermatids (Supplemental Figure S2A) and mature sperm (Supplemental Figure S2B).

### Generation of *Fam170a* and *Fam170b* KO mice using CRISPR/Cas9

**Figure 3:**
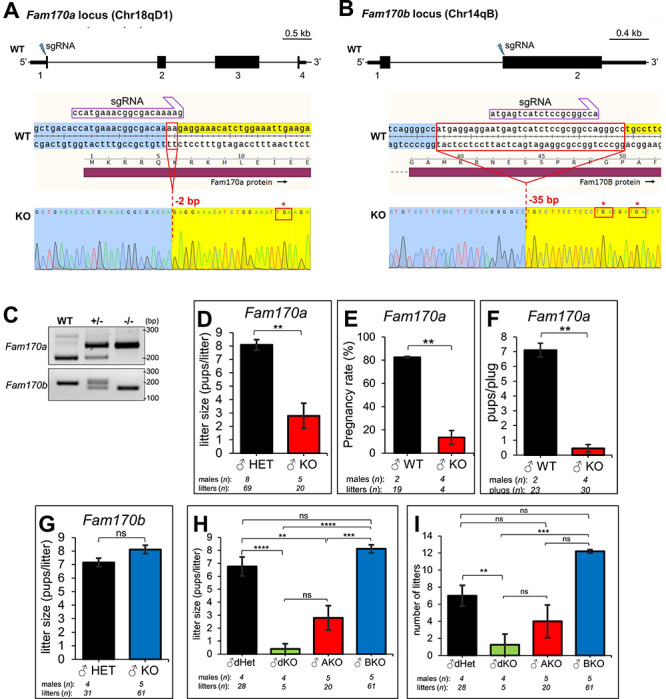
CRISPR/Cas9-mediated generation of *Fam170a^−/−^* and *Fam170b^−/−^* mice and fertility analysis. (A) *Fam170a* gene targeting strategy for null allele creation on chromosome 18. (B) *Fam170b* gene targeting strategy for null allele creation on chromosome 14. The location of the ORF is demarcated by the tall black boxes, while the short boxes signify the 5′ and 3′-UTR. (A and B) Sanger sequencing confirmation of the mutation with the sgRNA shown annealed to the WT reference sequence and the deleted nucleotides outlined in a red box. Resultant early stop codons are outlined in red boxes with an asterisk. (C) Genotyping PCR result for the *Fam170a* and *Fam170b* null alleles. WT, wildtype; +/−, heterozygous; −/−, KO. (D) Average litter sizes (pups/litter) from natural mating analysis for *Fam170a^+/−^* males (*n* = 8) mated to littermate controls and Fam*170a^−/−^* males (*n* = 5) mated to WT females for 6 months. (E) Average pregnancy rates (# litters/# copulation plugs) from timed-mating analysis for WT males (*n* = 2) and *Fam170a^−/−^* males (*n* = 4) mated to WT females. (F) Average pups/copulation plug from timed-mating analysis for WT males (*n* = 2) and *Fam170a^−/−^* (*n* = 4) males mated to WT females. (G) Average litter sizes from natural mating analysis for *Fam170b*^+/−^ males (*n* = 4) mated to littermate controls and *Fam170b*^−/−^ males (*n* = 5) mated to WT females for 6 months. (H) Average litter sizes from natural mating analysis for dHET males (*n* = 4) mated with littermate control females and dKO males (*n* = 4) mated with WT females. Data from Fam*170a^−/−^* males (AKO, red) and Fam*170b^−/−^* (BKO, blue) are reproduced here for statistical comparison. (I) Average number of litters sired per male for Fam*170a^−/−^* males (*n* = 5), *Fam170b^−/−^* males (*n* = 5), dHET males (*n* = 4), and dKO males (*n* = 4). Data represent means ± SEM. Analyzed by Student’s *t*-test for comparison of two groups and one-way ANOVA with Tukey-Kramer HSD post hoc testing for comparison of more than two groups. Asterisks indicate significance level: ^*^, *P* < 0.05; ^**^, *P* < 0.01; ^***^, *P* < 0.001; ^****^, *P* < 0.0001; ns, not significant.

To evaluate the consequences of systemic loss of the *Fam170a* and *Fam170b* genes independently, a sgRNA targeting the ATG start codon of the *Fam170a* ORF on the mouse chromosome Chr18qD1 was designed in silico (Figure 3A) and validated in vitro as reported previously [35]. For the *Fam170b* ORF on Chr14qB, a sgRNA was designed to target the 5′-end of exon 2 since this gene only contains two exons (Figure 3B). Electroporation of the sgRNAs into B6D2F1 zygotes and transfer to the oviducts of CD-1 mice for delivery resulted in a frameshift mutation and early stop codon introduction from a 2-bp deletion in *Fam170a*, which generated the *Fam170a^em1Osb^* allele (Figure 3A). A frameshift mutation resulting from a 35-bp deletion and early stop codon introduction was also achieved in *Fam170b*, which generated the *Fam170b^em2Osb^* allele (Figure 3B). The homozygous mutant (−/−) alleles were confirmed by PCR and Sanger sequencing (Figure 3A and B). In order to genotype the 2-bp deletion of the *Fam170a* KO, the strategy described by Gaudet [37] for allele-specific PCR genotyping of SNPs was adapted (Figure 3C). Using the available anti-FAM170B antibody, we were also able to confirm the loss of FAM170B protein in *Fam170b^−/−^* tissues. Testis cross-sections (Supplemental Figure S2A) and sperm from the cauda epididymis (Supplemental Figure S2B) of adult *Fam170b*^−/−^ males appropriately lost the signal for FAM170B compared with *Fam170b^+/−^* controls. To verify the *Fam170a* mutation, qRT-PCR was performed on RNA extracted from WT, *Fam170a^+/−^*, and *Fam170a^−/−^* adult male mice. It was found that an mRNA transcript is produced in *Fam170a^−/−^* mice and it is significantly upregulated (Supplemental Figure S3). To investigate the transcript sequences, primers were designed to amplify the nucleotide sequence corresponding to the 5′-UTR and the first 300 amino acids from WT and *Fam170a*^−/−^ mouse cDNA. Bidirectional Sanger sequencing of the respective 1156-bp amplicon of the WT and the 1154-bp amplicon of the *Fam170a*^−/−^ revealed that the *Fam170a*^−/−^ cDNA possesses the expected 2-bp deletion and subsequent frameshift, which introduces an early stop codon in exon 1 and causes alternative splicing around exon 2 that incorporates several base pairs of intron sequence (Supplemental Figure S3B). It is important to note that there are methionines in exon 3 that could theoretically serve as the new translation initiation site; however, if translation began from the most upstream methionine in exon 3, it would generate a polypeptide lacking first 114 amino acids of FAM170A and would lack the NLS, thereby preventing the aberrant protein from entering the nucleus (Supplemental Figure S3B).

### *Fam170a* ^−/−^ leads to male subfertility, while *Fam170b*^−/−^ males are fertile

To assess how the loss of the *Fam170a* and *Fam170b* genes impacts male fertility, −/− males were mated in a trio breeding scheme to WT females for 6 months of natural mating while recording litter sizes and number of litters sired over the period. Control matings of +/− males to +/− or −/− females were also setup for comparison and to demonstrate the normal fertility of females. In addition to evaluating the fertility of mutant mice, these breeding schemes were designed to maximize the number of mutant offspring for downstream studies and to reduce the total number of cages needed. Of the eight *Fam170a*^+/−^ control males included in the natural mating study, four were mated to *Fam170a*^+/−^ females and the other four were mated to *Fam170a*^−/−^ females to ensure that this testis-specific protein did not affect female fertility. The average litter sizes (pups/litter) from *Fam170a*^+/−^ males mated to *Fam170a*^−/−^ females were comparable (7.4 ± 0.6) to *Fam170a*^+/−^ males mated to *Fam170a*^+/−^ females (8.8 ± 0.3) (data not shown). After 6 months of mating, *Fam170a*^−/−^ males mated to WT females produced a significantly reduced average litter size (2.8 ± 0.9) when compared with *Fam170a*^+/−^ control males (8.1 ± 0.4) (Figure 3D). To further characterize the subfertility of *Fam170a*^−/−^ males, timed matings with WT females were conducted in a trio breeding scheme, counting a total of at least 20 copulation plugs between all males included for either WT or *Fam170a*^−/−^ genotypes. *Fam170a* WT males produced successful pregnancies (litters/copulation plugs) for 82.6 ± 0.8% of copulation plugs, while *Fam170a*^−/−^ males only led to 13.5 ± 5.9% of pregnancy success (Figure 3E). The *Fam170a*^−/−^ males also sired significantly fewer pups/plug (0.5 ± 0.3) compared with WT males (7.5 ± 0.5) (Figure 3F). In contrast, *Fam170b*^−/−^ males mated to WT females produced average litter sizes (8.1 ± 0.3) comparable to *Fam170b*^+/−^ males mated to *Fam170b*^−/−^ females (7.2 ± 0.3), underscoring that this model had no impact on male fertility (Figure 3G).

To investigate the possibility of functional redundancy or partial compensation between *Fam170a* and *Fam170b*, the mutant lines were crossed to yield mice heterozygous for both loci (dHET) and homozygous for both loci (dKO). To evaluate the fertility of the new mutants, dKO males were mated in trios to WT females and dHET males were mated to dHET female litter mates. After 6 months of natural mating, dKO males led to reduced litter sizes (0.4 ± 0.4) compared with dHET controls (6.8 ± 0.7) (Figure 3H). In addition, the average number of litters sired per male was reduced among dKO males (1.25 ± 1.25) compared with dHET males (7.0 ± 1.2) (Figure 3I). When comparing dKO males to *Fam170a^−/−^* or *Fam170b^−/−^* males, it is important to note that there were no statistical differences in the litter sizes (Figure 3H) or litter numbers (Figure 3I) between *Fam170a*^−/−^ males and dKO males, suggesting that the *Fam170b* does not play a compensatory role with *Fam170a*.

### *Fam170a* ^−/−^ mice exhibit abnormal spermiogenesis and sperm head morphology

**Figure 4:**
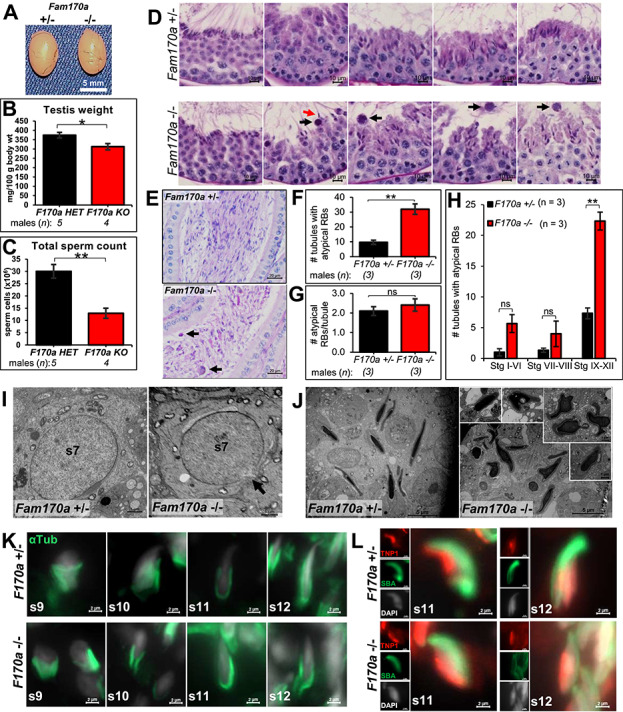
Histological characterization of *Fam170a^−/−^* consequences during spermatogenesis. (A) Gross image of testis from an age matched adult Fam*170a^+/−^* and *Fam170a^−/−^* mice. (B) Average testis weight (wt) normalized to 100-g body weight ([mg absolute testis wt/g body weight] × 100) for Fam*170a^+/−^* (*n* = 5) and *Fam170a^−/−^* (*n* = 4) mice. (C) Average total cauda epididymal sperm count from Fam*170a^+/−^* and *Fam170a^−/−^* mice by CASA. (D) PAS-Hematoxylin staining of testis cross-sections showing representative images from stage VIII to XII seminiferous tubules to highlight spermiation and spermatid elongation from Fam*170a^+/−^* and *Fam170a^−/−^* mice. Black arrow indicated atypical RBs. Red arrow indicated retained spermatids. (E) PAS-Hematoxylin staining of corpus epididymis sections from Fam*170a^+/−^* and *Fam170a^−/−^* mice. Black arrows indicate abnormal epididymis luminal contents. (F) Quantification of number of seminiferous tubules containing large atypical RBs from counting 219 tubules from *Fam170a^+/−^* mice (*n* = 3) and 221 tubules from *Fam170a^−/−^* mice (*n* = 3). (G) Quantification of the number of atypical RBs found per affected tubule in *Fam170a^+/−^* and *Fam170a^−/−^* mice. (H) Quantification of the number of seminiferous tubules containing atypical RBs stratified by spermatogenic stage in *Fam170a^+/−^* and *Fam170a^−/−^* mice. (I) STEM of step 7 round spermatids from *Fam170a^+/−^* and *Fam170a^−/−^* testis. Black arrow indicates posterior nuclear membrane defect. (J) STEM of elongated spermatids from *Fam170a^+/−^* and *Fam170a^−/−^* testis. Insets show various nuclear malformations in *Fam170a^−/−^* testis. (K) Immunofluorescent staining of manchette microtubules in testis cryosections from *Fam170a^+/−^* and *Fam170a^−/−^* testis from stages IX to XII tubules. Anti-α-Tubulin antibody was used to label manchette microtubules (green) and counterstained with DAPI (gray) to label nuclei. (L) Immunofluorescent subcellular localization of TNP1 (red) to identify issues with histone-to-protamine transition in spermatid chromatin condensation. SBA (green) is used to label the acrosome and DAPI (gray) counterstain to label nuclei. Data represent means ± SEM. Analyzed by Student’s *t*-test. Asterisks indicate significance level: ^*^, *P* < 0.05; ^**^, *P* < 0.01; ^***^, *P* < 0.001; ^****^, *P* < 0.0001; ns, not significant.

To characterize the cause of subfertility in the *Fam170a^−/−^* males, a gross analysis of the testis was performed, and sperm counts were acquired. The testis sizes showed no discernable differences between *Fam170a^+/−^* and *Fam170a^−/−^* males (Figure 4A), and absolute testis weights were similar for *Fam170a^−/−^* (115.7 ± 6.0 mg) and *Fam170a^+/−^* (113.4 ± 4.0 mg) controls. However, there was a significant difference in body weights (*P* = 0.04), as *Fam170a^−/−^* males weighed more (37.0 ± 1.9 g) than *Fam170a^+/−^* males (30.3 ± 0.5 g). This difference led to a significant difference in the relative testis weights (normalized to 100-g body weight), as *Fam170a^−/−^* males were less (313 ± 17 mg) than *Fam170a^+/−^* males (374 ± 14 mg) (Figure 4B). Additionally, sperm counts extracted from the cauda epididymis of *Fam170a^−/−^* males were reduced (12.9 ± 2.1 × 10^6^ cells) by more than 50% of *Fam170a^+/−^* control males (30.1 ± 2.8 × 10^6^ cells) (Figure 4C). We next investigated the testis and epididymis histology using PAS-hematoxylin staining according to published guides [38, 49, 50]. Representative images depicting the complete progression of spermatogenesis from stage I to stage XII seminiferous tubules are presented in Supplemental Figure S4 for *Fam170a^+/−^* males and in Supplemental Figure S5 for *Fam170a^−/−^* males. Striking defects in spermiation, the coordinated process of mature spermatid release into the seminiferous tubule lumen [51], and spermatid elongation were detected in *Fam170a^−/−^* mice. Although step 16 spermatids were found lining the lumen of stage VIII tubules in preparation for release in *Fam170a^−/−^* mice, step 16 spermatids were found still attached to the lumen in the following stage IX tubules, and large atypical residual bodies (RBs) were present at the luminal surface rather than being phagocytosed by the Sertoli cells (Figure 4D). These atypical RBs were found persisting in the lumen of the successive stages X, XI, and XII. In addition to the defective spermiation, *Fam170a^−/−^* mice exhibited a delayed or abnormal spermatid elongation between stages IX and XII. The nuclei of *Fam170a^−/−^* spermatids did not become thin and elongated until stage XII, compared with the nuclei of *Fam170a^+/−^* spermatids which became thin and dense-staining by stage XI (Figure 4D). Histological examination of the corpus epididymis revealed a lumen filled with normal sperm in *Fam170a^+/−^* mice, while the epididymis of *Fam170a^−/−^* mice presented with several large, round cellular material that correlated with the atypical RBs found in the testis (Figure 4E). Atypical RBs can be a frequent background finding in mice; however, a significant increase in their appearance can be indicative of impaired spermatid cytoplasm removal, impaired formation of the tubulobulbar complex and removal of apical ectoplasmic specializations (apical ES), or Sertoli cell dysfunction [50, 52]. Since the appearance of the large atypical RBs was a substantial defect in the *Fam170a^−/−^* mice, the number of atypical RBs and stages of appearance was quantified after counting at least 200 seminiferous tubules each across *Fam170a^−/−^* and *Fam170a^+/−^* mice. *Fam170a^−/−^* mice presented with a significantly greater number of tubules containing atypical RBs in the lumen (32.0 ± 3.5) compared with *Fam170a^+/−^*controls (9.7 ± 1.5) (Figure 4F); however, the number of RBs per tubule was no different between *Fam170a^−/−^* (2.4 ± 0.3) and *Fam170a^+/−^* (2.1 ± 0.2) control mice (Figure 4G). Finally, most of the atypical RBs appeared following spermiation in stages IX–XII, with a significantly higher number of affected tubules in the *Fam170a^−/−^* mice (22.3 ± 1.5) than in the *Fam170a^+/−^*controls (7.3 ± 0.9) (Figure 4H).

To look at the ultrastructural differences in *Fam170a^−/−^* mice, TEM analysis of testis sections was used to evaluate spermatids. *Fam170a^−/−^* step 7 round spermatids displayed a peculiar apparent break in the nuclear membrane opposite the acrosome (Figure 4I). TEM analysis of elongated condensing spermatids in *Fam170a^+/−^*testis revealed normal nuclear shape and acrosome formation; however, spermatids in *Fam170a^−/−^* testis have variable and remarkably abnormal nuclear shapes despite a present acrosome compartment (Figure 4J). While appearing dense, many of these spermatids exhibited perforations the chromatin, budding off of the nuclei, and some substantial gaps between the spermatid and Sertoli cell plasma membranes (Figure 4J, insets). To investigate the cause of abnormal head shaping in *Fam170a^−/−^* spermatids, testis cryosections were stained with an antibody for α-tubulin to label the manchette microtubules. The manchette microtubules in *Fam170a^−/−^* elongating spermatids from step 9 to step 12 (Figure 4K) presented normally, despite obvious head-shape defect at step 10 and less extensive elongation in step 12 when compared with *Fam170a^+/−^* spermatids. Due to the nuclear-shape defects in *Fam170a^−/−^* spermatids highlighted by TEM analysis and immunostaining, the early stages of nuclear compaction were investigated by staining testis cryosections with an antibody for Transition protein 1 (TNP1) [53]. Both *Fam170a^+/−^* and *Fam170a^−/−^* mice showed positive staining for TNP1 in the nuclei of step 11 and step12 when co-stained with SBA to label the acrosome and counterstained with DAPI to label nuclei (Figure 4L). However, as *Fam170a^+/−^* step 11 spermatids exhibited normal TNP1 subcellular localization to the anterior region of the nucleus which spread to the posterior region in step 12, TNP1 subcellular localization remained at the base of the nucleus in *Fam170a^−/−^* step 11 and step 12 spermatids (Figure 4L). TNP1 expression was not detected in the nucleus of spermatids after stage VI in either *Fam170a^+/−^* or *Fam170a^−/−^* testis, suggesting that the temporal expression of TNP1 was normal according to the report by Zhao [53] in both conditions (data not shown).

**Figure 5:**
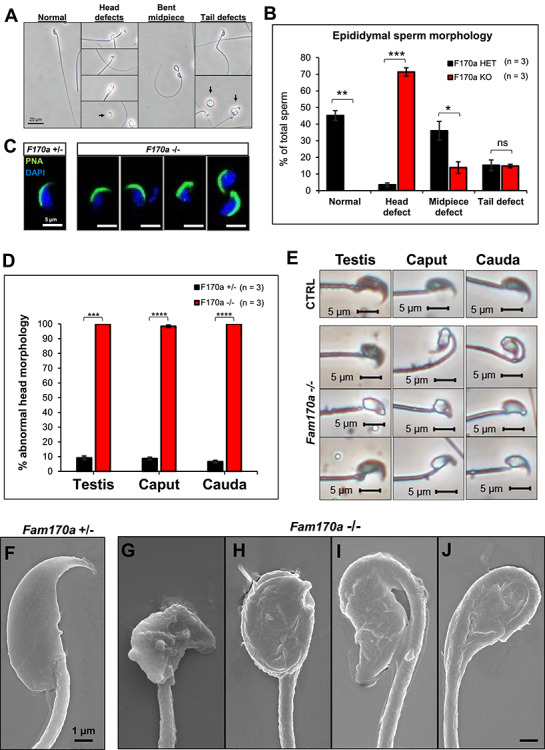
Comparison of sperm head morphology differences between *Fam170a^+/−^* and *Fam170a^−/−^* spermatozoa. (A) Representative phase contrast images of sperm abnormal morphology populations found in *Fam170a*^+/−^ and *Fam170a^−/−^* cauda epididymal sperm isolates. Black arrows indicate the sperm morphology defect being identified when multiple sperm appear in the image. (B) Quantification of sperm abnormal morphology populations in *Fam170a*^+/−^ (*n* = 3) and *Fam170a^−/−^* (*n* = 3) cauda epididymal sperm isolates, counting at least 300 individual spermatozoa for each genotype. (C) Immunofluorescent staining of the acrosome status of sperm isolated from the cauda epididymis of *Fam170a*^+/−^ and *Fam170a^−/−^* mice. Acrosomes are labeled with PNA-FITC (green) and nuclei are counterstained with DAPI (blue). (D) Quantification of abnormal head morphology comparing testicular, caput epididymal, and cauda epididymal spermatozoa from *Fam170a*^+/−^ and *Fam170a*^−/−^ mice. At least 80 testicular, 150 caput epididymal, and 400 caudal epididymal spermatozoa were counted across the mice in each genotype (E). Representative images of sperm head morphology from *Fam170a*^+/−^ (CTRL) and *Fam170a*^−/−^ mice extracted from the testis, caput epididymis, or cauda epididymis. (F) *Fam170a^+/−^* spermatozoa displaying normal head morphology by SEM. (G—J) Fam*170a^−/−^* spermatozoa exhibiting various head and connecting piece abnormalities, such as amorphous heads (G and H) or bent neck (I and J).

For *Fam170b^−/−^*mice, testis assessment (Supplemental Figure S6A–C) and epididymal spermatozoa (Supplemental Figure S6D–G) were normal, thus ending the study of this model. However, to further investigate the abnormal head morphology in *Fam170a^−/−^* mice, the morphology of mature spermatozoa from the cauda epididymis was evaluated. A series of phase contrast images of epididymal spermatozoa from *Fam170a^+/−^* and *Fam170a^−/−^* mice were captured to quantify the percentages of normal spermatozoa and sperm with head, midpiece, or tail abnormalies, as reported by others [39–41]. Remarkably, none of the spermatozoa from *Fam170a^−/−^* males exhibited normal morphology, as all sperm from these mice exhibited abnormal head morphology such as amorphous head shape, bent neck, and decapitation (Figure 5A and B). Though sperm neck defects are often grouped with midpiece defects for abnormal morphology classification [54], bent neck and decapitation were grouped together with other head abnormalities, similar to the report by Bruner-Tran [40], because they indicate an issue with the head-tail coupling apparatus (HTCA) [55] and bent neck is a defect commonly found with the loss of proteins involved in nuclear condensation [56]. It was found that 71.4 ± 2.5% of *Fam170a^−/−^* sperm presented with head defects as the only abnormality, compared with 3.5 ± 1.1% of *Fam170a^+/−^* controls (Figure 5B). The head defects observed in *Fam170a^−/−^* sperm could be further subdivided into amorphous heads only (34.6 ± 3.5%), bent neck (33.2 ± 4.3%), and decapitation (3.6 ± 0.9%). In addition to the head defects, some *Fam170a^−/−^* spermatozoa displayed a bent midpiece or tail defect; however, these defects were minor when compared with *Fam170a^+/−^* spermatozoa controls. Due to this head formation defect, the status of the acrosome in mature spermatozoa was investigated since the acrosome is essential for spermatid head shaping [57]. *Fam170a^−/−^* spermatozoa possessed an intact acrosome structure, although the acrosome shape was thickened or distorted along with the sperm head-shape abnormalities (Figure 5C). To investigate whether any aberrant morphology originated during spermatogenesis or during epididymal transit, spermatozoa released from the testis, caput epididymis, and cauda epididymis were compared by phase contrast microscopy. Congruent with the spermatogenesis defects detailed in Figure 4D for *Fam170a^−/−^* mice, all spermatozoa exhibited abnormal head morphology that originated in the testis (Figure 5D), while the bent neck appeared during transit through the caput epididymis and persisted in the cauda epididymis (Figure 5E). Scanning electron microscopy images were taken of cauda epididymal sperm from *Fam170a^+/−^* and Fam*170a^−/−^* mice for detailed observation of the sperm head malformations. Compared with the normal sperm head morphology of *Fam170a^+/−^* mice (Figure 5F), the amorphous heads (Figure 5G and H) and bent necks (Figure 5I and J) of the *Fam170a^−/−^* sperm were clearly captured.

Human and mouse FAM170A were suggested by Lei [15] and Xu [16] to be transcription factors, with the later stating that FAM170A regulates gene expression at heat shock element (HSE) sequences in the genome [58]. To investigate whether there was an evidence for this in vivo in mice, qRT-PCR was conducted to look at the expression of Heat shock genes regulated by HSE that are essential for male fertility (Supplemental Figure S7A). There were no changes in the expression of any Heat shock gene in *Fam170a^−/−^* testis compared with *Fam170a^+/−^* tissues. To look at other targets of FAM170A potential transcriptional regulation that could account for the defects in spermiation and head shaping in *Fam170a^−/−^* mice, several genes essential for nuclear compaction (Supplemental Figure S7B) [48], components of apical ectoplasmic specializations (Supplemental Figure S7C) [59], and components of the nuclear envelop LINC complexes (Supplemental Figure S7D) [60]. Loss of *Fam170a* did not significantly alter the expression of any of the genes tested.

### *Fam170a* ^−/−^ sperm exhibit diminished progressive motility

**Figure 6:**
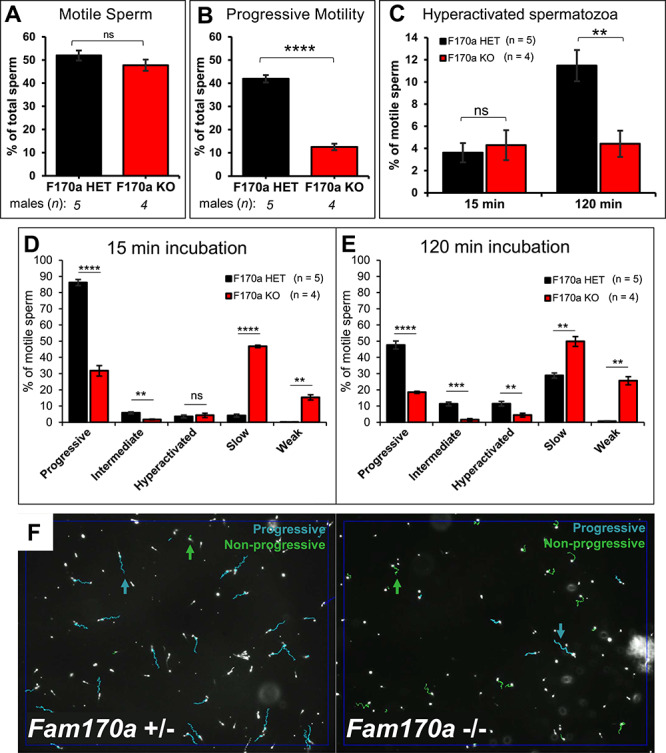
*Fam170a* ^+/−^ and *Fam170a*^−/−^ sperm motility analysis and comparison by CASA and CASAnova. (A) Total sperm motility measured by CASA for Fam*170a*^+/−^ (*n* = 5) and *Fam170a^−/−^* (*n* = 4) sperm. (B) Progressive motility represented as a percent of total sperm counted by CASA for Fam*170a*^+/−^ and *Fam170a^−/−^* sperm. (C) Sperm hyperactivation classified by CASAnova in Fam*170a*^+/−^ and *Fam170a^−/−^* sperm following release from the cauda epididymal tissue at 15 min and after in vitro capacitation (120-min incubation). (D–E) CASAnova classification of sperm motility populations in *Fam170a*^+/−^ and *Fam170a^−/−^* sperm (D) after release from the cauda epididymal tissue at 15 min and (E) after 120 min incubation in capacitation media. (F) Representative images of CASA analysis from *Fam170a*^+/−^ and *Fam170a^−/−^* spermatozoa differentiating progressive (teal) and nonprogressive (green) motility tracks. Arrows identify individual sperm tracks. Data represent means ± SEM. Analyzed by Student’s *t*-test. Asterisks indicate significance level: ^*^, *P* < 0.05; ^**^, *P* < 0.01; ^***^, *P* < 0.001; ^****^, *P* < 0.0001; ns, not significant.

Due to the striking morphological abnormalities in *Fam170a^−/−^* sperm, sperm motility and kinematics were assessed as a measure of mature function using Computer-Assisted Sperm Analysis (CASA). The total percentage of motile sperm was comparable between *Fam170a^+/−^* (52.0 ± 2.1%) and Fam*170a^−/−^* (47.8 ± 2.4%) spermatozoa (Figure 6A). The percent of progressively motile sperm, however, was significantly reduced for *Fam170a^−/−^* mice (12.6 ± 1.4%) compared with *Fam170a^+/−^* controls (42.0 ± 1.6%) (Figure 6B). For evaluation of sperm hyperactivation, a requirement for sperm capacitation and fertilization [61–63], spermatozoa were incubated in capacitation medium for a total of 120 min and the machine learning algorithm CASAnova was used to classify motility patterns based on kinematic measurement outputs from CASA, as described in Goodson [45]. CASAnova analysis classified motile sperm into groups of either vigorous (progressive, intermediate, or hyperactive) or nonvigorous movement (slow or weak) at the initial time point of 15-min incubation in capacitation medium and after 120 min. The percent of hyperactivated motile *Fam170a^+/−^* sperm increased as expected after 120-min incubation to 11.5 ± 1.4%, while *Fam170a^−/−^* sperm hyperactivation failed to increase (4.4 ± 1.2%) (Figure 6C). In comparison of all five motility classifications by CASAnova, most motile *Fam170a^+/−^* sperm exhibited progressive motion (86.2 ± 1.9%) at 15 min which dwindled to slow motility after 120 min, while the percentage of intermediate and hyperactive sperm increased (Figure 6D and E). Conversely, most motile *Fam170a^−/−^* sperm exhibited slow motion (46.9 ± 0.8%) and many were weak (15.4 ± 1.6%) at 15 min (Figure 6D), which became worse after 120 min (Figure 6E) and failed to show an increase in intermediate or hyperactive motility. Representative images from CASA analysis demonstrate far fewer progressive motility tracks (labeled blue) and many more nonprogressive motility tracks (labeled green) in *Fam170a^−/−^* spermatozoa when compared with *Fam170a^+/−^* spermatozoa (Figure 6F).

## Discussion

The FAM170A and FAM170B proteins are the only two members of the Family with sequence similarity 170 and have previously unknown biological importance. Reports published by Lei [15] and Xu [16] for FAM170A (also named ZNFD for its C2H2 ZnF domain) and a report by Li [17] for FAM170B established that these proteins were testis-specific and provided preliminary evidence that these proteins may have a role in male reproduction. The reports for FAM170A used in vitro cell culture assays in HeLa and COS-7 cells to demonstrate that recombinant human FAM170A localizes to the nucleus and used reporter gene assays to establish that FAM170A acts as a transcriptional activator of the *JUN* proto-oncogene [15] and regulates the transcriptional activity at HSE sequences in the genome of COS-7 cells [16]. Combined with data from the Human Protein Atlas that places the subcellular localization of FAM170A in the nucleus of spermatocytes of human testis samples, these data suggested that the human *FAM170A* gene may be important in male fertility. The report for FAM170B [17] used a commercial antibody to resolve its subcellular localization to the acrosome of round spermatids and mature spermatozoa. The authors also pretreated mouse sperm with the antibody to block FAM170B function and found that anti-FAM170B-treated sperm displayed a reduced capacity to fertilize oocytes and a reduced capacity to undergo acrosome reaction in vitro [17]. Finally, the authors used a coimmunoprecipitation (Co-IP) experiment to indicate that FAM170B interacts with Golgi-associated PDZ and coiled-coil motif-containing protein (GOPC) [17], a protein essential for acrosome formation and fertility [64]. These data, taken together, allowed reasonable speculation that FAM170B could also be important for male fertility.

In this report, we demonstrate the high conservation of FAM170A in eutherian mammals and the wide conservation of FAM170B in the three subclasses of mammals and a species of reptile. These data suggest that FAM170B is an older protein and that FAM170A likely arose to address a new function for eutherians [65]. This is corroborated by the discordant temporal expression of *Fam170a* and *Fam170b* in the mouse (Figure 1E), as the expression of *Fam170a* coincides with the appearance of pachytene spermatocytes, while *Fam170b* expresses after the appearance of round spermatids during the first wave of seminiferous epithelium [26]. Despite both proteins having a C2H2 ZnF, only FAM170A has an NLS and demonstrates protein localization to the nucleus, both with in vitro expression of recombinant protein in COS-7 cells and in our *Fam170a^cFlag^* KI mouse model. Although FAM170B is an acrosome protein, it is not unprecedented for an acrosome protein to possess DNA-binding domains [66, 67]. One of the most striking findings from this study is that despite all of the preliminary evidence suggesting that FAM170B could be important for male fertility, we found that *Fam170b* is expendable for male fertility in mice and its loss has no discernable effect on the acrosome (Supplemental Figure S2), spermatogenesis, or sperm morphology (Supplemental Figure S6).

Here, we discovered that KO of *Fam170a* leads to male subfertility. Upon observing the reduced litter sizes produced by Fam*170a*^−/−^ males in natural mating experiments, timed matings were setup to better characterize the degree of subfertility. The timed mating experiment provided evidence that mating behavior was normal in Fam*170a*^−/−^ males and that the subfertility was instead due to markedly reduced pregnancy rates and a decrease in average pups born per copulation plug. *Fam170a* and *Fam170b* dHET and dKO mice were generated by crossing the mutant lines together in order to examine whether the subfertility in Fam*170a*^−/−^ mice was attributed to partial compensation by the WT copy of *Fam170b*. Upon the observation that there were no statistical differences in the average litter size or in the average number of litters produced between *Fam170a*^−/−^ males and dKO males, the hypothesis that *Fam170b* has a compensatory relationship with *Fam170a* was rejected.

The cause of subfertility in Fam*170a*^−/−^ males was investigated using histological analysis of the testis and epididymis, as well as cytological and functional characterization of spermatozoa. In *Fam170a*^−/−^ testis, large atypical RBs were found in the lumens of stage IX through stage XII tubules and elongated spermatids were found still attached to Sertoli cells in some stage IX tubules. These late-stage spermatogenesis defects are indicative of improper spermatid cytoplasm removal and defective spermiation (disengagement of mature spermatids from Sertoli cell ectoplasmic specializations) [50, 52]. These atypical RBs are likely the explanation for the abnormal round cellular material found in the lumen of the *Fam170a*^−/−^ epididymis, as this is also a hallmark of defective spermatid cytoplasm removal during spermiation [52]. The reduction in normalized testis weight and sperm count in *Fam170a*^−/−^ mice is also defects commonly associated with spermiation defects [51]. More remarkably, sperm extracted from the testis and epididymis of Fam*170a*^−/−^ mice displayed abnormal head morphology, with amorphous head shape and sperm bent at the neck region (after entering the caput epididymis) being most apparent. These defects highlight an issue with spermatid head shaping and potentially the integrity of the HTCA of the connecting piece during spermiogenesis, as detailed in several reviews [51, 52, 55, 68, 69]. Additionally, the bent neck abnormality (also termed head-bent-back in other literature) is commonly reported with the loss of genes involved in nuclear compaction, such as *Tnp2* [70], *Prm1* [71], *Camk4* [72], and *H1f7* [73] or with the loss of genes involved in nucleocytoplasmic transport required during spermiation [56], such as *Spem1* [74]. The posterior nuclear envelope defects present in *Fam170a*^−/−^ round spermatids by TEM closely resembled a similar issue described in the mouse KO of the *H1f7* nuclear compaction gene [73], while the severe nuclear malformations in *Fam170a*^−/−^ elongated spermatids were reminiscent of the defects described in KOs of the *Chd5* [75] nuclear condensation gene and the *Cetn1* [76] basal body-nucleus connection gene. These similarities suggest that FAM170A may serve roles related to chromatin condensation in the spermatid nucleus and may explain the altered subcellular localization of TNP1 in *Fam170a*^−/−^ step 11 spermatids that appeared at the base of the nucleus rather than the anterior. The aberrant spermatid head shape and elongation in *Fam170a*^−/−^ testis could not be explained by an issue with manchette microtubes, as this structure appeared normal throughout spermatid elongation steps. Other structures impacting spermatid head shape, such as the perinuclear ring or apical ES, may be attributed [52, 69, 77].

Despite the striking sperm head morphology defects in Fam*170a*^−/−^ sperm, however, the acrosome develops into an intact structure in mature spermatozoa, providing evidence that FAM170A does not play a role in acrosome formation. Finally, we found markedly impaired progressive motility in Fam*170a*^−/−^ sperm. While the number of motile sperm in *Fam170a*^−/−^ mice was comparable to *Fam170a*^+/−^ controls, most *Fam170a*^−/−^ sperm exhibited slow or weak motility classifications by CASA and CASAnova. It was reviewed by Lehti [55] that defects in the connecting piece often lead to decreased progressive motility. Additionally, reduced sperm motility is often found with the loss of genes involved in nuclear compaction and shaping [73, 75], and thus, the poor progressive motility in *Fam170a*^−/−^ spermatozoa could be attributed to the abnormal head morphology. Overall, the subfertility in Fam*170a*^−/−^ males is characterized by abnormal spermiation, reduced sperm count, abnormal sperm head morphology, and poor sperm progressive motility. Xu [16] suggested that FAM170A regulates transcription at HSE sequences in the genome based on in vitro assays, which prompted us to test whether the transcription of key heat shock genes or other genes governing relevant processes in spermatogenesis were altered in *Fam170a*^−/−^ mice. We found that the loss of FAM170A did not affect the expression of any heat shock proteins essential to spermatogenesis, genes governing nuclear compaction, components of apical ES, or components of nuclear LINC complexes (nuclear envelope bridging proteins) in our qRT-PCR assay (Supplemental Figure S7). Though we did not find evidence of transcriptional changes mediated by FAM170A, it does not rule out the possibility that FAM170A is a transcription factor, as it could be in control of other genes. Alternatively, FAM170A may interact with these proteins directly or indirectly to mediate its in vivo function rather than through transcriptional control. However, at this time, the molecular functions of FAM170A are unknown.

Since mutations in the human *FAM170A* are identified on the National Center for Biotechnology Information Single Nucleotide Polymorphism Database (dbSNP) (www.ncbi.nlm.nih.gov/snp), the results reported herein have implications for human male infertility. Future studies investigating the correlation between *FAM170A* mutations and human subfertility or infertility have the potential to link idiopathic cases of infertility to a new gene.

In conclusion, we demonstrated that *Fam170a* is important for male fertility, as this gene is required for proper spermiation at the end of spermatogenesis, sperm head morphology, and sperm progressive motility. Future studies into FAM170A should investigate the molecular functions of the protein, and although no validated antibody for the mouse protein exists, our *Fam170a^cFlag/cFlag^* mouse model may be useful in future proteomic studies. These studies into the molecular role of FAM170A could uncover new genes with important roles in male fertility and identify potential contraceptive target proteins.

## Conflict of interest

The authors have declared that no conflict of interest exists.

## Supplementary Material

MS_REVISED_Fig_S1_ioaa082

MS_REVISED_Fig_S2_ioaa082

MS_REVISED_Fig_S3_ioaa082

MS_REVISED_Fig_S4_ioaa082

MS_REVISED_Fig_S5_ioaa082

MS_REVISED_Fig_S6_ioaa082

MS_REVISED_Fig_S7_ioaa082

MS_REVISED_Table_S1_ioaa082
