# Supplementary material for: Knockout of family with sequence similarity 170 member A (Fam170a) causes male subfertility, while Fam170b is dispensable in mice
Source: Biol Reprod. 2020 May 22;103(2):205–22. doi: 10.1093/biolre/ioaa082 (PMC7401401; doi:10.1093/biolre/ioaa082)
Supplement: MS_REVISED_Fig_S1_ioaa082 [file ms_revised_fig_s1_ioaa082.pdf]

SeqNLS Prediction Results

Prediction result (The predicted NLS(s) are underlined)

FAM170A-iso1:

<u>MKRRQKRK</u>HLEIEESKEAGISKSQEDISHPESTGVPKAQSPGVG
EVSSASEYFSCVSSPQKLIHRSKGTWKLLQDSSKPRSPLDQVP
EGEATTAPSQQASSSCPSYKTCVSSLCMNKEERGMKIYYMQVQ
MKKGVAISWDTKETSESLEKQPRMEEATLPEGVWVGTPPSDVS
TRNLLSDSEPIGEEKEHEEKPESDSPPGSPAVEERPRAKTPDW
LVTMENGFRCMACCRVFATMESLQEHVQYGIREGFSCHVFHLT
MAQLIGSMESESTQEEEEDHTEETEKPKEEKAEEQQPTEEDVG
MKKPWSQCPGCVFDSPKDRRRRKDHCDNSGS

score range	color
0.1 - 0.3	
0.3 - 0.5	
0.5 - 0.7	
0.7 - 0.8	
0.8 - 0.86	
0.86 - 0.89	
> 0.89	

The predicted NLS(s) (score cutoff = 0.86)

UniProt Protein ID	Predicted NLS	Start	Stop	The highest score of matches within the prediction
Q66LM6	MKRRQKRK	1	8	0.903
