# Supplementary figures and images for: Knockout of family with sequence similarity 170 member A (Fam170a) causes male subfertility, while Fam170b is dispensable in mice
Source: Biol Reprod. 2020 May 22;103(2):205–22. doi: 10.1093/biolre/ioaa082 (PMC7401401; doi:10.1093/biolre/ioaa082)

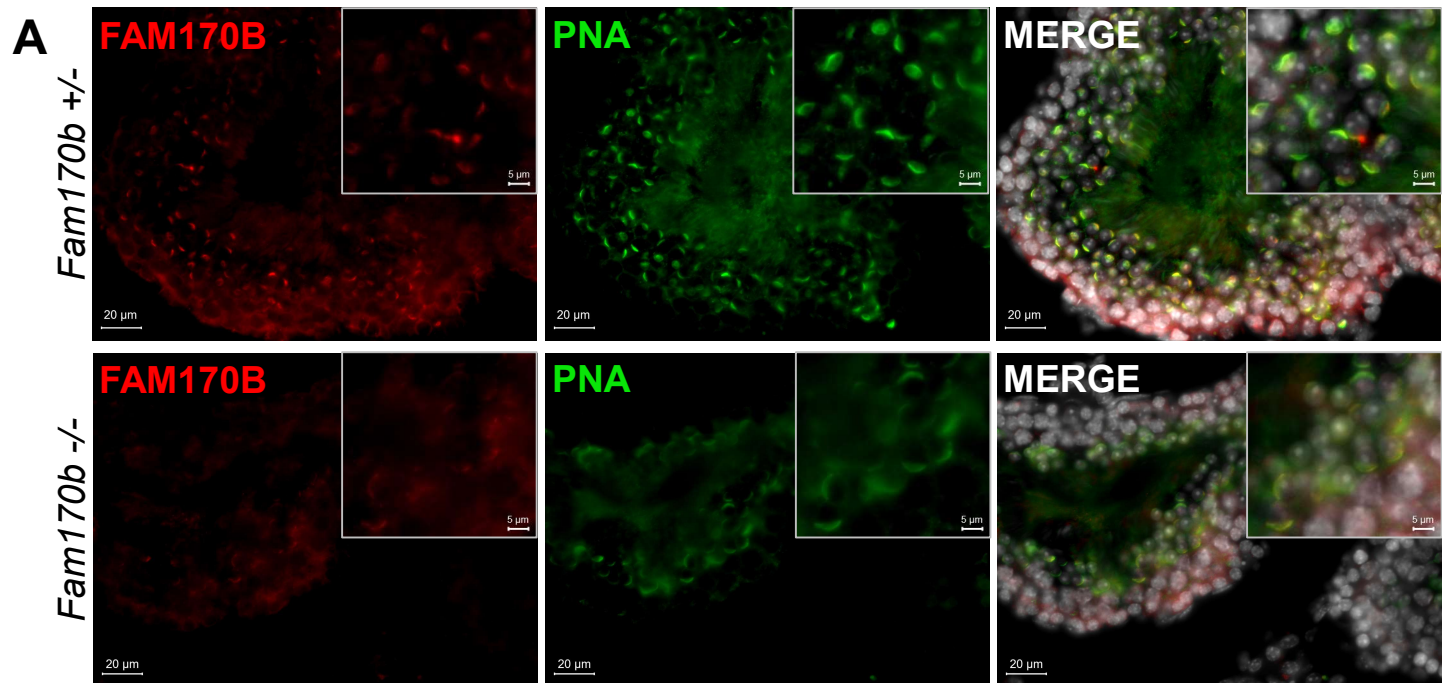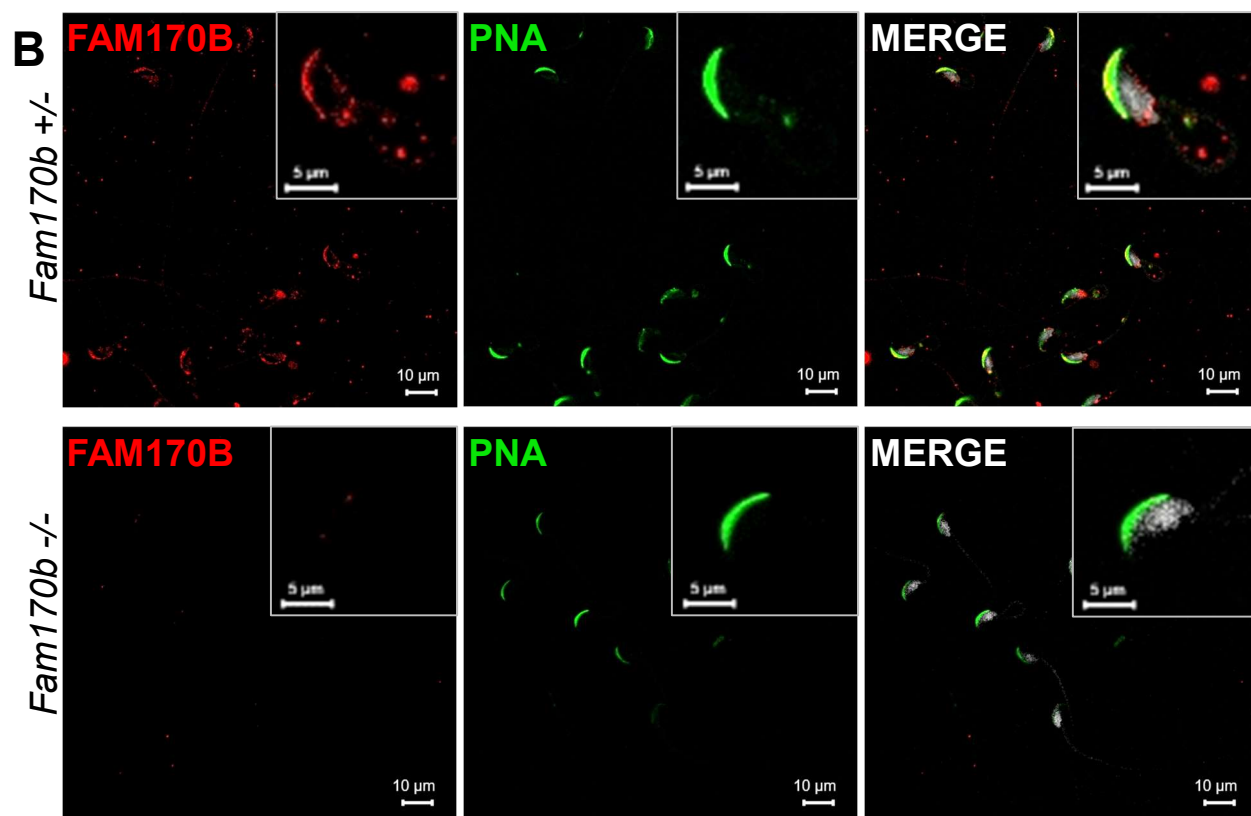

Supplement: MS_REVISED_Fig_S2_ioaa082 [file ms_revised_fig_s2_ioaa082.pdf]

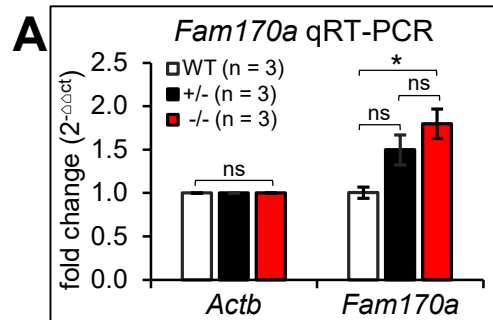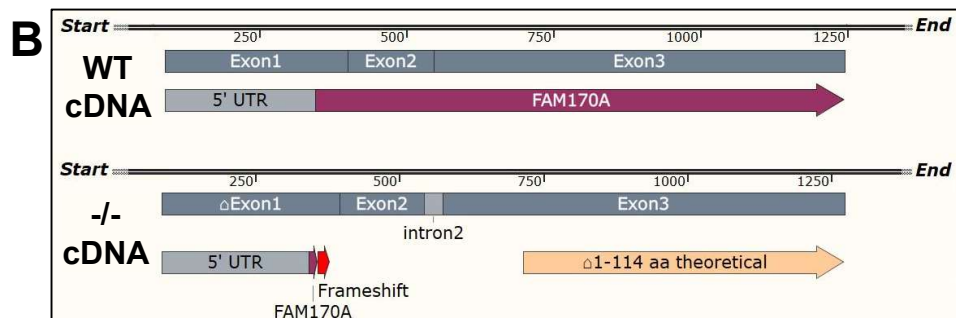

Supplement: MS_REVISED_Fig_S3_ioaa082 [file ms_revised_fig_s3_ioaa082.pdf]

*Fam170a* +/-

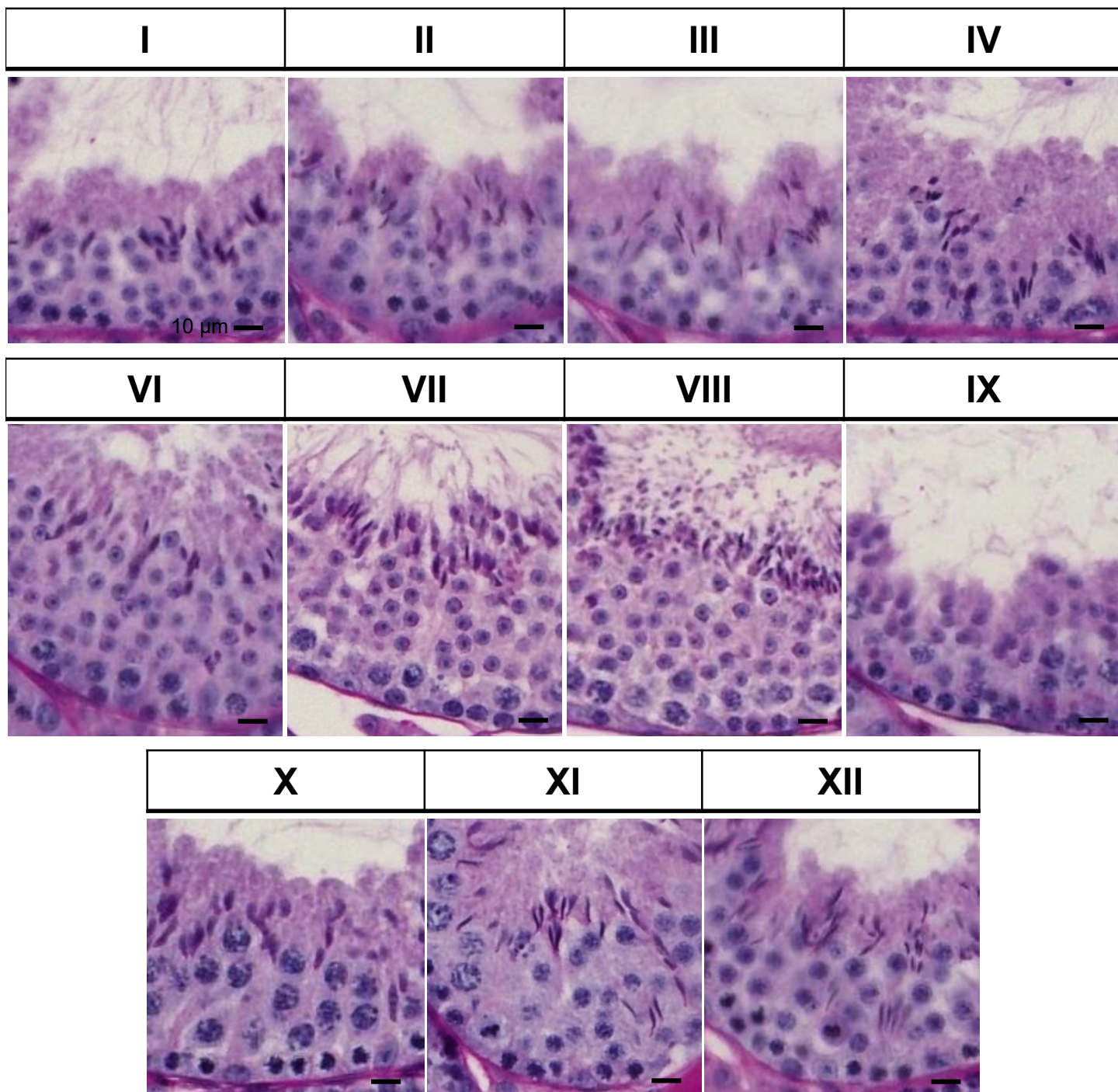

Supplement: MS_REVISED_Fig_S4_ioaa082 [file ms_revised_fig_s4_ioaa082.pdf]

# *Fam170a* KO

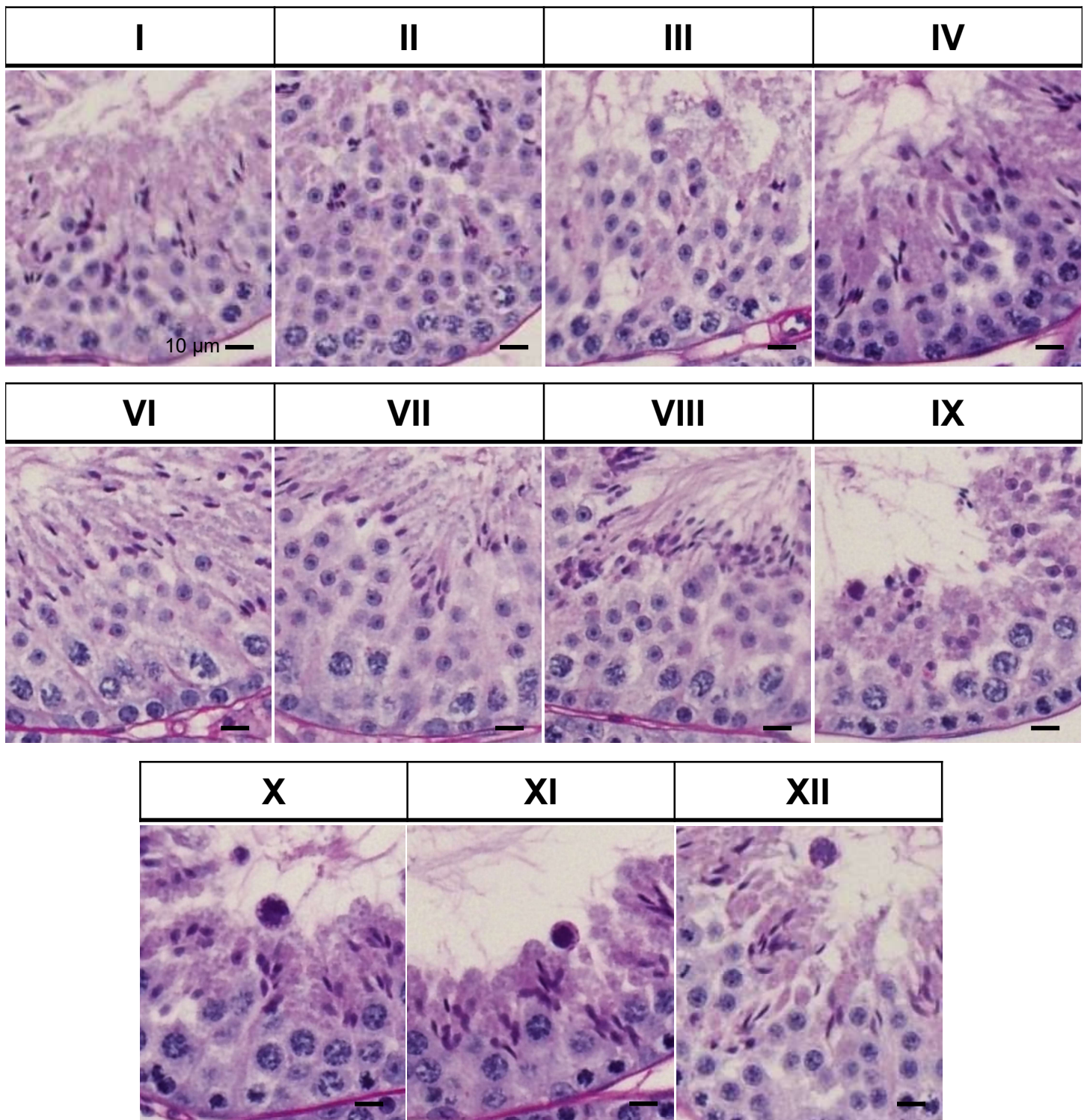

Supplement: MS_REVISED_Fig_S5_ioaa082 [file ms_revised_fig_s5_ioaa082.pdf]

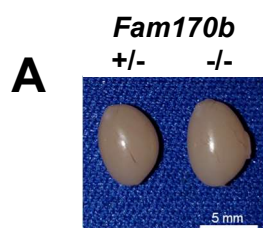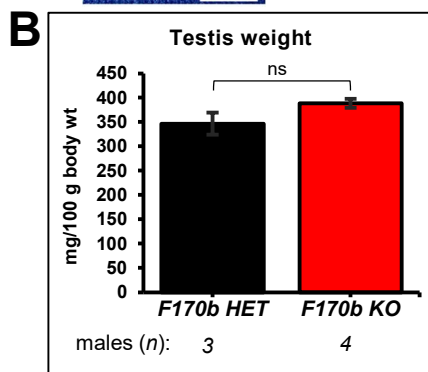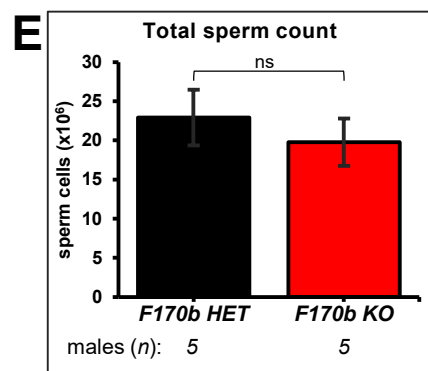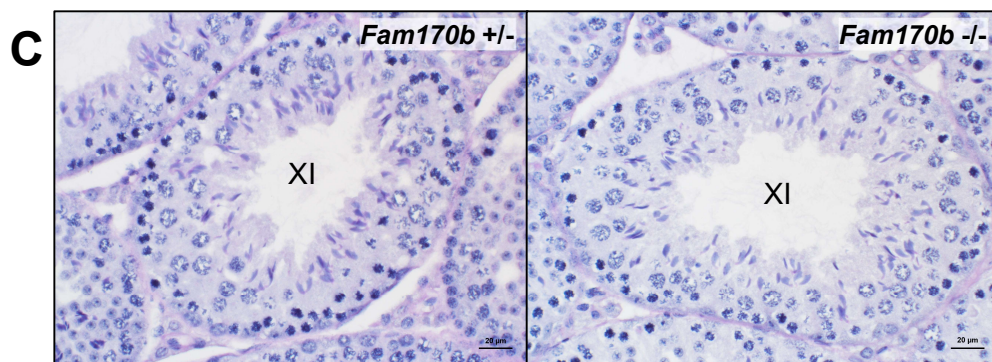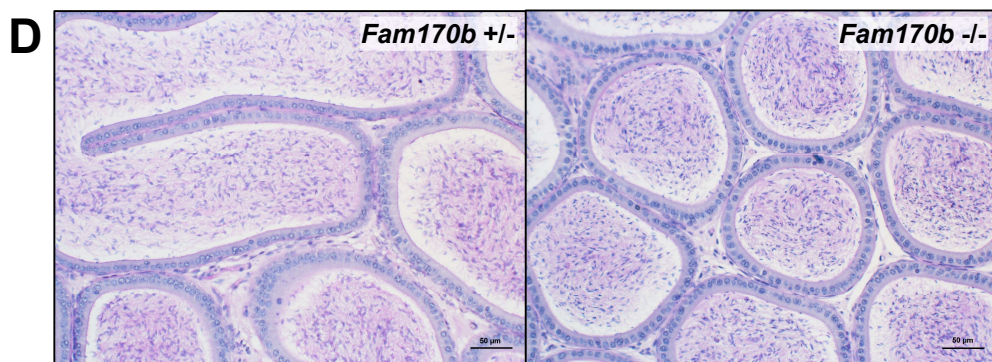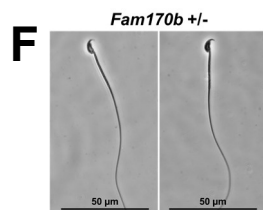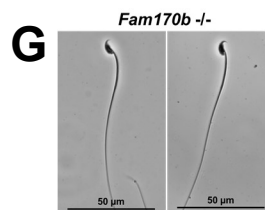

Supplement: MS_REVISED_Fig_S6_ioaa082 [file ms_revised_fig_s6_ioaa082.pdf]

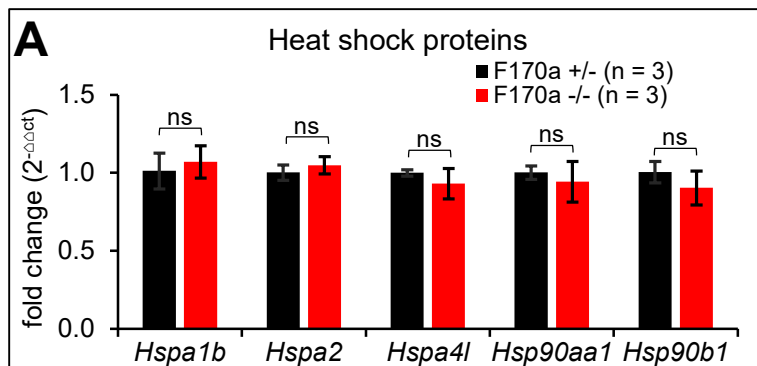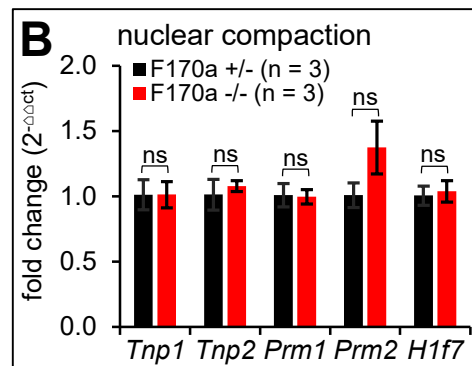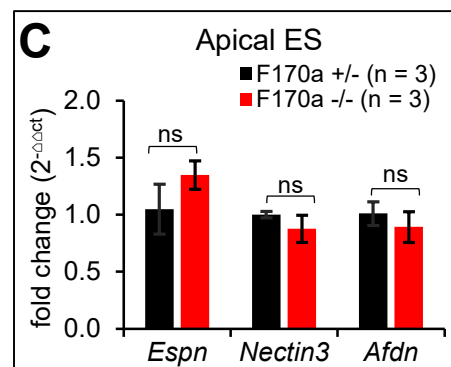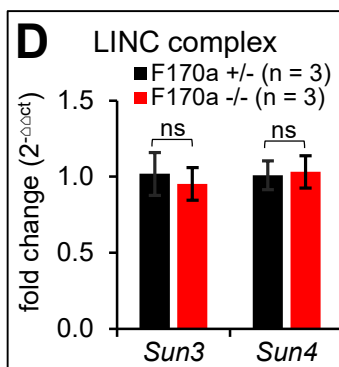

Supplement: MS_REVISED_Fig_S7_ioaa082 [file ms_revised_fig_s7_ioaa082.pdf]
