# Supplementary material for: Knockout of family with sequence similarity 170 member A (Fam170a) causes male subfertility, while Fam170b is dispensable in mice
Source: Biol Reprod. 2020 May 22;103(2):205–22. doi: 10.1093/biolre/ioaa082 (PMC7401401; doi:10.1093/biolre/ioaa082)
Supplement: MS_REVISED_Table_S1_ioaa082 [file ms_revised_table_s1_ioaa082.pdf]

Supplemental Table 1. Primers used for qRT-PCR in this study

Primer ID:	Sequence (5' - 3')	Amplicon
mActB-F1	AAGTGTGACGTTGACATCCG	222 bp
mActB-R1	GATCCACATCTGCTGGAAGG	
mF170a-F7	TGAAGAGTCAAAGGAAGCTGGA	174 bp
mF170a-R7	CAGGTTCCCTTACTACGATGGA	
mHSPA1B-F1	AAAGGAGCCTCTGGCTCAGG	121 bp
mHSPA1B-R1	GTAATCGGTGCCCAAGCAGC	
mHSPA2-F1	TCGGCTGAGCAAAGACGACA	145 bp
mHSPA2-R1	CTCGTCTTCCACGGTCTGCT	
mHSPA4L-F1	TGCGTTGCAGTGTGCGATTC	131 bp
mHSPA4L-R1	AGACTTCACATTCCCCAGTCCC	
mHSP90AA1-F1	GCTTTCAGAGCTGTTGCGGT	129 bp
mHSP90AA1-R1	TGGTCCTTGGTCTCACCTGT	
mHSP90B1-F1	AGGCCCTCAAGGACAAGATAGA	137 bp
mHSP90B1-R1	GCCCGTCTGGTATGCTTGTG	
mTNP1-F1	CGACCAGCCGCAAGCTAAAG	145 bp
mTNP1-R1	TTGCGACTTGCATCATCGCC	
mTNP2-F1	ACACTCACCTGCAAGACCCA	134 bp
mTNP2-R1	GCCCTGAGCTACGCCTCTTA	
mPRM1-F1	GCACCATGGCCAGATACCGAT	123 bp
mPRM1-R1	GACGGCAGCATCTTCGCC	
mPRM2-F1	CCACCACCACCACAGACACA	139 bp
mPRM2-R1	TCGGGATCTTCTGCAGCCTC	
mH1F7-F1	GCCAGGGATGAAGCCAGGAT	144 bp
mH1F7-R1	TACTGGCCTCTCGGGTTCCT	
mSUN3-F1	GGTTCCCAGGGTCACATCCT	150 bp
mSUN3-R1	ACATTTCTTCATAACGCCATAGACAGAG	
mSUN4-F1	ACCGTCTGGATCTTCTGTAGTTTTG	122 bp
mSUN4-R1	GCACCTGAAAGAGGAGGCTCA	
mESPN-F1	GGAGAGGGAGCAGAAGCGAA	138 bp
mESPN-R1	AAGATGACCTGTCGCTGCCA	
mNECTIN3-F1	TGTGGTGCCTTAGCTGGATCA	149 bp
mNECTIN3-R1	ACAGTCTGTGTACTTTTGCCATGT	
mAFDN-F1	TGAAGTGCATGTCAGTGGAGAAA	137 bp
mAFDN-R1	CCTTCTTGGCAGGAATGGCG	
